# Correlative evidence for co-regulation of phosphorus and carbon exchanges with symbiotic fungus in the arbuscular mycorrhizal *Medicago truncatula*

**DOI:** 10.1371/journal.pone.0224938

**Published:** 2019-11-11

**Authors:** Jan Konečný, Hana Hršelová, Petra Bukovská, Martina Hujslová, Jan Jansa

**Affiliations:** 1 Department of Experimental Plant Biology, Faculty of Science, Charles University, Viničná, Prague, Czech Republic; 2 Institute of Microbiology, Czech Academy of Sciences, Vídeňská, Prague, Czech Republic; University of Michigan, UNITED STATES

## Abstract

Research efforts directed to elucidation of mechanisms behind trading of resources between the partners in the arbuscular mycorrhizal (AM) symbiosis have seen a considerable progress in the recent years. Yet, despite of the recent developments, some key questions still remain unanswered. For example, it is well established that the strictly biotrophic AM fungus releases phosphorus to- and receives carbon molecules from the plant symbiont, but the particular genes, and their products, responsible for facilitating this exchange, are still not fully described, nor are the principles and pathways of their regulation. Here, we made a *de novo* quest for genes involved in carbon transfer from the plant to the fungus using genome-wide gene expression array targeting whole root and whole shoot gene expression profiles of mycorrhizal and non-mycorrhizal *Medicago truncatula* plants grown in a glasshouse. Using physiological intervention of heavy shading (90% incoming light removed) and the correlation of expression levels of *MtPT4*, the mycorrhiza-inducible phosphate transporter operating at the symbiotic interface between the root cortical cells and the AM fungus, and our candidate genes, we demonstrate that several novel genes may be involved in resource tradings in the AM symbiosis established by *M*. *truncatula*. These include glucose-6-phosphate/phosphate translocator, polyol/monosaccharide transporter, DUR3-like, nucleotide-diphospho-sugar transferase or a putative membrane transporter. Besides, we also examined the expression of other *M*. *truncatula* phosphate transporters (*MtPT1-3*, *MtPT5-6*) to gain further insights in the balance between the "direct" and the "mycorrhizal" phosphate uptake pathways upon colonization of roots by the AM fungus, as affected by short-term carbon/energy deprivation. In addition, the role of the novel candidate genes in plant cell metabolism is discussed based on available literature.

## Introduction

Research on the arbuscular mycorrhizal (AM) symbiosis is mainly driven by the urgency to design more sustainable agricultural production systems for the future, of which the AM fungi are supposed to be an important component [1]. Yet the progress is still limited by a number of factors, besides others by incomplete understanding of the molecular principles and their regulation of mycorrhizal functioning at cellular, organismal, and ecosystem levels [2–4].

The arbuscular mycorrhizal symbiosis (AMS) is arguably one of the oldest and the most widespread inter-kingdom associations on Earth [5] with a strong impact on the physiology and ecology of their plant hosts including many crop plants [6], as well as functioning and stability of the entire ecosystems [7, 8]. In the times of rapidly changing environmental conditions, the influence of AM fungi on plant drought-, salt- or pathogen tolerance is growing in importance ([9, 10], and references therein). Yet, we are still only scratching surface of full understanding of the economy of the symbiosis, i.e., the rates of commodity exchanges between the symbiotic partners, and cellular responses in the host plants to the symbiosis, particularly with respect to genes or proteins directly involved in metabolism and transfer of carbon (C) molecules towards the fungal partner [11]. Because the AM fungus receives about 2–20% of plant C [12], such knowledge seems important for understanding the physiology of AMS and C allocation in plants, as well as in understanding ecosystem processes particularly at times of high demand for increasing both the production and sustainability of agricultural systems (United Nations Sustainable Developmental Goals). This could be achieved either by direct application of AM fungi in agriculture, by utilization of indigenous AM fungal communities, or by valorization of specific „know-how”on metabolic pathways established in the AMS for improvement of crop yields and/or quality [13, 14].

Regarding phosphorus exchanges between the symbiotic partners, the situation appears rather simple. Plant can absorb phosphorus in the form of orthophosphate (P), mainly as dihydrogen phosphate, H_2_PO_4_^-^, directly from the soil solution via phosphate transporters (PT) located at the surface of the rhizodermal cells (and the root hairs protruding from them), with subsequent generation of P-depletion zones in the soil around the roots. This is so called “direct P uptake pathway”[15]. To avoid the emerging P deficiency in plants increasingly exploiting P in the immediate vicinity of their roots, while relying solely on the direct P uptake pathway, a "mycorrhizal (indirect) pathway" is often established by many plant species [16]. The P has repeatedly been shown to be the major commodity transferred from the AM fungi towards the host plant, involving specific genes responsible for this pathway in the periarbuscular membrane (PAM) of colonized root cells (arbuscocytes). In *Medicago truncatula* Gaertn., *MtPT4* was revealed as the AM-specific PT [17–19], which is crucial for functional AMS in this plant species [20], making this gene one of the reference genes for established and functional AMS. Out of six PT described from the *M*. *truncatula* genome, *MtPT1-MtPT6* (from here as PT1-PT6), it is the only mycorrhiza-specific P transporter. Similarly, a single or multiple AM-specific PTs do exist in other plant species: *LjPT3* in *Lotus japonicus* L. [21], *OsPT11/13* in *Oryza sativa* L. [22, 23], *PhPT4* in *Petunia × atkinsiana* (Sweet) D. Don ex W. H. Baxter [24], *NtPT5* in *Nicotiana tabacum* L. [25], *StPT3/4/5* in *Solanum tuberosum* L. [26, 27], *LePT4/5* in *Solanum lycopersicum* L. [27] or *HvPT8* in *Hordeum vulgare* L. [28]. During AMS, the expression of such AM-specific PTs is usually highly upregulated, and consequently, the PT involved in the "direct pathway" tend to be downregulated [25, 29]. Yet, despite the extensive knowledge on P transfer from the AM fungus to the plant, much less is actually known about the mechanisms behind the reduced C transfer from the plant to the symbiotic AM fungi.

Biotrophic AM fungus acquires the C solely from the host plant–although the exact forms of C/energy as well as the exchange rates of C for P are still a matter of debate [30]. The most common hypothesis is a "sugar pathway": Export of sucrose (Suc) to periarbuscular space is followed by its cleavage by means of acid invertases into glucose (Glc) and fructose (Fru), of which the Glc can be taken up by the fungal saccharide transporter (ST). This hypothesis is strongly supported by expression pattern and enzymatic activity of Suc cleaving enzymes, mainly the cell wall-bound acid invertases [31, 32]. An old ST-candidate for C export from the plant cell was a Mtst1 [33], but recently it has been shown that MtSWEET1b may play a role in Glc flow to the arbuscule [34]. Furthermore, the substrate specificity of AM fungal ST (*RiMST2*, *RiMST4* and *RiMST5*; [35, 36]), its expression in arbuscules and importance in formation of specific symbiotic structures such as arbuscules, supports the uptake of Glc and its relevance as one of the major forms involved in C transfer from the plant to the fungus. But the remaining Fru is generally not a substrate for those ST, so the fate of Fru is still unknown–leaving the question open about whether this form of C is taken back to the plant cells [1].

More recent experimental evidence established the transfer of fatty acids towards the fungus as alternative pathway for shuffling the C from the plant to the AM fungus. Higher production of fatty acids by the plants upon mycorrhization is a logic consequence of AMS establishment, because the arbuscocytes have a high demand for new cell membranes since the PAM is larger than the plasmatic membrane [37]. Yet, the fatty acids may also represent the other form of reduced C molecule transferred across periarbuscular space, as demonstrated recently [38]. Some key molecular players in this "lipid pathway" were already revealed, based on forward genetic screening of AM-defected mutants, and isotopolog profiling [39]. Notably, this cross-kingdom lipid transfer seems to play a major role for the physiology of the biotrophic fungus, since the dysfunction of lipid transfer prevents completion of its life cycle by disrupted vesicle formation, reduced exploration of the root volume and incomplete development of extraradical mycelia and spores [39–45]. This may relate to loss of cytoplasmic fatty acid synthetase genes, as demonstrated for the *Rhizophagus* genome [46].

Given the generally fragmentary knowledge about the molecular mechanisms of C transfer from the plant to the AM fungus, we describe here a *de novo* effort to identify genes potentially involved in the symbiotic C transfer in the AMS, asking specific following questions:

Which (novel) genes encoding ST–with particular focus on those ST showing mycorrhiza-specific transcriptional induction–can play a role in the C transfer from *M*. *truncatula* towards *Rhizophagus irregularis* (Blaszk., Wubet, Renker & Buscot), one of the model organisms in AMS research?

Is there a transcriptional regulation when changing the availability of C for the plant, i.e., upon shading treatment, and shading-related alterations in the P uptake pathways?

To achieve these goals, two pot experiments were carried out, in which the plants were grown for 35, 49, 63 (Exp 1); and 67 and 71 days (Exp 2), respectively, and where strong but only a short-term shading was applied or not on the mycorrhizal and non-mycorrhizal plants harvested as two timepoints after shading onset. Expression of genes was measured either by using Affymetrix microarrays or by specific quantitative real-time PCR assays. In addition to ST, we also measured the transcription of the different PT of the *M*. *truncatula* in both shoots and roots, and compared the expression patters of genes involved in both mycorrhizal P and C fluxes.

## Materials and methods

### Plants and growth conditions

**Fig 1:**
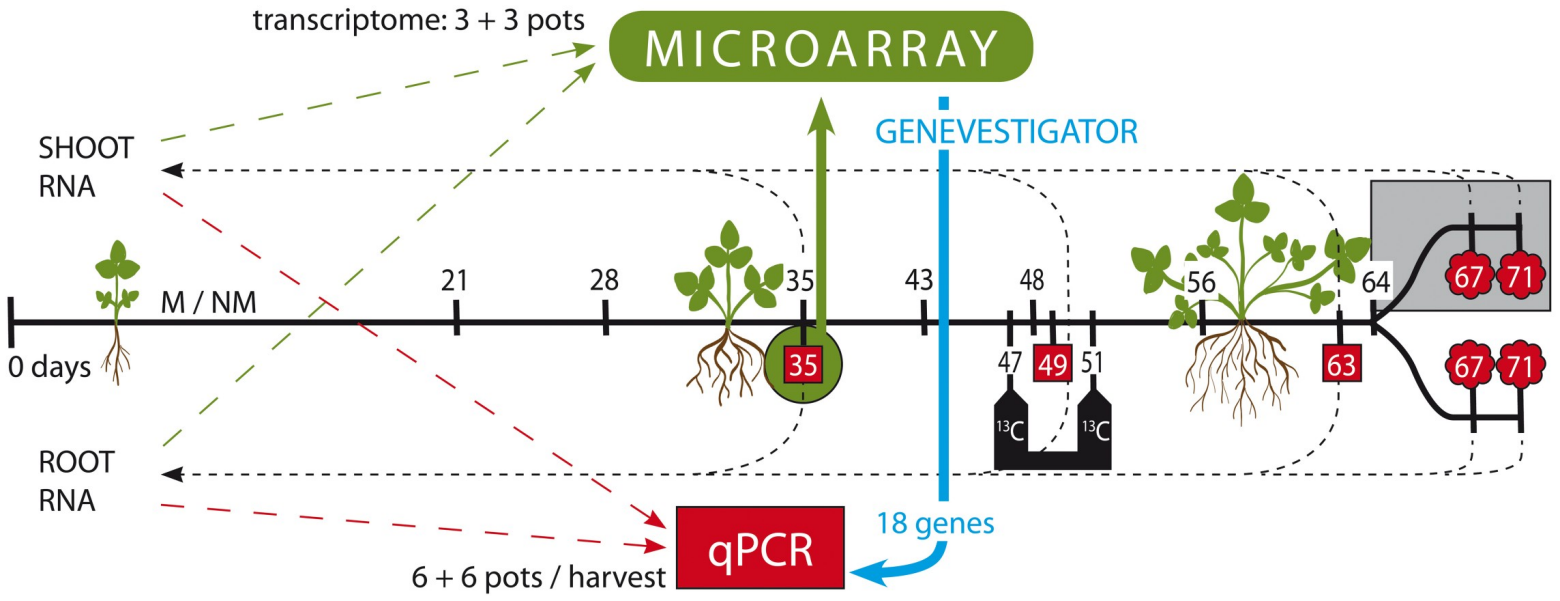
The scheme of both experiments. In Exp 1, the pots for plant gene expression profiling were harvested at 35, 49 or 63 days post planting (dpp), red squares (n = 6). Whole shoot and whole root total RNA of 6 mycorrhizal (M+) and 6 non-mycorrhizal (NM) pots was isolated from plants collected at each harvest. From the 35 dpp harvest, the transcriptomes were generated (green, n = 3). Then, expression of 18 genes (of those, 11 genes were newly selected via Genevestigator—blue) was studied using specific qPCR assays, using samples from all the harvests (red areas). From paralell pots, physiological parameters were measured (black numbers, n = 3) and ^13^C labeling was carried out (white numbers in the black box, n = 7). In Exp 2, 12 M+ and 12 NM pots were included, of which a half was shaded starting at 64 dpp, and all the pots were harvested for whole root and whole shoot total RNA extraction at 67 or 71 dpp (i.e., 3 or 7 days after shading onset; red clouds, n = 3).

We conducted two pot experiments (Exp 1 and Exp 2), using the same pot setup and glasshouse cultivation conditions, with the exception of the growing season and length of plant cultivation (for details, see below). Barrel medic (*M*. *truncatula* Jemalong J5; seeds originally provided by Gérard Duc from INRA, Dijon, France, and subcultured for several generations in the lab) seeds were scarified and surface-sterilized with 98% sulfuric acid for 20 min, rinsed thoroughly with distilled water and sown on a wet filter-paper in room temperature. Two-days-old seedlings were planted in 2.5 L pots (3 or 4 seedlings per pot in Exp 1 or Exp 2, respectively) containing a mixture of autoclaved quartz sand (grain size < 3 mm), autoclaved zeolite MPZ 1–2.5 mm (Zeopol, Břeclav, Czech Republic) and gamma-irradiated (> 25 kGy) LT soil (volumetric ratio 45:45:10; for soil characteristics see Řezáčová et al. [47], for the characteristics of the planting mixture see Püschel et al. [48]). The substrate was added with rhizobial suspension and with mycorrhizal or nonmycorrhizal inocula (for their description, please see below). The pots were placed in a glasshouse at the Institute of Microbiology (Czech Academy of Sciences, Prague, Czech Republic) at random positions with average temperature of 25°C or 28°C, respectively (S1 Table). Light was a combination of natural light and supplemental high pressure metal halide lamps (providing a minimum photosynthetic flux density of 150 μmol m^-2^ s^-1^ at plant level) to compensate for low natural light intensity and to extend the photoperiod to 14 h (S1 Table). Plants were watered daily with distilled water and fertilized weekly (starting 22 days post planting—dpp) with 65 mL of Long-Ashton solution with reduced phosphorus content (to 20% of the original value, i.e., 0.26 mM P [49]). In Exp 1, the plants grew from December to February and they were harvested as described below at 35, 49, 63 dpp for the gene expression profiling (n = 6), with additional harvests for root colonization, biomass production and P content (see supplementary information for details). In Exp 2, the plants grew from March to June and half of the pots were placed under dark-green shading cloth (canopy), allowing just 10% of the incoming light to pass through (S1 Photos). Shading lasted for 3 or 7 days, after which period of time the pots were harvested (i.e., after 67 and 71 dpp; n = 3; see Fig 1).

### Rhizobial and mycorrhizal inocula

Rhizobial suspension of *M*. *truncatula*-compatible *Sinorhizobium meliloti* strain 10 (isolated from the LT soil [48]) was added to all pots before sowing (300 μL per planting hole/seedling, containing approximately 2 x 10^9^ cells). All the plants had well-developed nodules at the end of experiments (numbers and/or vitality of the nodules were not quantified).

Mycorrhizal inoculum was obtained from pot cultures of an AM fungus *R*. *irregularis* strain SYM5 (described in Gryndler et al. [50]), which were incubated in the glasshouse for 8 months prior to the experiments described here, with leek (*Allium porrum* L.) as a host plant. Leek roots from the inoculum pots were harvested and cut mechanically into small pieces (< 1 cm) and then mixed back into the potting substrate from the inoculum production pots (which was the same as the potting substrate used in the experiments described here). This material (90 g) was mixed with 900 mL of the potting substrate mixture and such mycorrhizal (M+) inoculum was placed in the upper half of each of the M+ pots, so the pots in the M+ treatments contained approximately 5% (v:v) of the mycorrhizal inoculum per pot. Non-mycorrhizal (NM) pots were prepared in the same way, but the NM inoculum (also called mock inoculum) was added to the NM pots instead of the M+ inoculum. NM inoculum was devoid of the AM fungus but produced for the same period of time under the same conditions as the M+ inoculum. Absence of mycorrhizal fungus (spores and substrate hyphae) in the NM inoculum-pots was confirmed microscopically before application to the relevant pots.

### Plant harvest and RNA isolation

The plants for RNA extraction from both experiments were harvested in the same way, but the time of harvest was between 11 a.m. and 12 a.m. and between 1 p.m. and 2 p.m. in Exp 1 and Exp 2, respectively. All plants in one pot were processed as a single unit. First, the shoots were cut at the hypocotyl-root boundary and immediately frozen in liquid nitrogen. Thereafter, the roots were quickly recovered from the substrate, cleaned by shaking under running cold tap-water, then the whole root system was blotted against paper towel and immediately frozen in liquid nitrogen. Plant samples were subsequently ground in mortar (while kept frozen at all times) and then subjected to RNA isolation or powderized samples further stored frozen at -80°C.

Frozen and pulvenized plant tissue–be it shoots or roots (approximately 3 g)–was mixed with 4 mL of phenol and 4 mL of extraction buffer (1.95 g of 100mM Tris, 0.0424 g of 100mM LiCl, 0.372 g of 10mM EDTA and 1 g of 1% SDS per 100 mL of DEPC-treated water (0.1% DEPC), adjusted to pH = 6), pre-heated to 80°C, eight zirconium (ZrO_2_) beads were then added and the mixture was vigorously vortexed. This was followed by 20 s incubation at 80°C in a water bath, followed by 40 s vortexing, and mixing with additional 4 mL of chloroform. The mixture was then centrifuged (20 min, 0°C, 5000 g) and supernatant was carefully recovered. Addition of chloroform and centrifugation (purification steps) were repeated four-times. Pure supernatant (2 mL) was mixed with 0.68 mL of 8M LiCl. Samples were precipitated on ice overnight and then centrifuged (20 min, 0°C, 20000 g). The pellet was resuspended in 0.5 mL of 75% EtOH (ice-cold). The samples were centrifuged again, the pellets then dissolved in 250 μL of RNase-free water, 500 μL of 96% ethanol and 25 μL of 3M sodium acetate. Samples were then precipitated on ice for 1 hour, then they were centrifuged and the pellets were dissolved in 100 μL of RNase-free water. Resulting total RNA was frozen in liquid nitrogen and stored at -80°C.

### DNase treatment and reverse transcription

To remove DNA from the total RNA samples, DNA-free^™^ DNA Removal Kit (Ambion, USA) was used following the instructions of manufacturer. To synthetize complementary DNA strand, Transcriptor High Fidelity cDNA Synthesis Kit (Roche, Switzerland) was used following the instructions of manufacturer, but using both primer types served at once: 2 μL of Random Hexamer Primer (600 pmol/μL) + 1 μL Anchored-oligo[dT]18 Primer (50 pmol/μL). A maximum of 4 μg total RNA was used as template. Resulting complementary single-strand DNA (cDNA) was stored at -20°C.

### Gene expression analyses on Affymetrix

Total RNA of roots and shoots of three M+ and three NM pots, grown for 35 days, was subjected to transcriptome-wide gene expression analysis using commercial Affymetrix microarrays for *M*. *truncatula*. The analyses were carried out in one of the European certified Affymetrix core lab (http://core.img.cas.cz) using GeneChip^™^ Medicago Genome Array (Applied Biosystems^™^, USA). The RNA quality was checked prior to the analyses by RNA-gel electrophoresis (S1 Fig) and Agilent 2100 Bioanalyzer (S2 Fig) and raw data were then curated and uploaded to Genevestigator^®^ 4-36-0 (Nebion, Switzerland) database [51] under the number MT-00071. Those experimental data were also deposited in NCBI's Gene Expression Omnibus [52] and are freely accessible through GEO Series accession number GSE126833 (https://www.ncbi.nlm.nih.gov/geo/query/acc.cgi?acc=GSE126833).

Application "Samples" within the Genevestigator platform was used to search through by key-words ("sugar", "transmembrane", "transporter", "saccharide", "monosaccharide", "sucrose", "sucrose synthase", "symporter", "antiporter", "alpha beta", "glucosidase", "glucose", "glycan", "fructose", "fructan", "trehalose", "sorbitol", "mannitol", "mannose", "mannan", "ascorbate", "cellulose", "sweet", "invertase", "sucrase") and also to seek through the published lists of ST (JCVI Medicago truncatula annotation database [53, 54], for details see S2 Table). Ten genes returning clearly visible changes in their expression levels in roots or in shoots between M+ and NM plants were manually selected and briefly analyzed for their relevance in saccharide transport or metabolism. One reference gene (*TIF2Fα*) was identified using "RefGenes" application within the Genevestigator software [55].

### Primer design

For analysis of gene expression by quantitative real-time PCR (qPCR) of the phosphate transporters *PT1* through *PT6* and previously used *TEF1α* reference gene, the published primer sequences were used [56, 57], see S2 Table. For 10 novel candidate genes selected from the Microarray analyses, and the novel reference gene *TIF2Fα*, the qPCR primers were designed using AlleleID 6.0 (Premier Biosoft, USA; see S2 Table). Target sequences of primers were 100% identical to the target sequences of corresponding Probe Sets of GeneChip^®^ Medicago Genome Array. The primers were subsequently synthetized and HPLC purified by Generi Biotech (Hradec Králové, Czech Republic).

### Quantitative PCR

Quantitative real-time PCR reactions composed of 13.2 μL of water, 0.4 μL of forward primer (25 μM), 0.4 μL of reverse primer (25 μM), 2 μL of cDNA template and 4 μL of 5× EvaGreen^®^ mastermix (Solis BioDyne, Tartu, Estonia). Reaction conditions were as follows: Initial denaturation at 95°C for 15 min, then 50 cycles of 95°C for 10 s, annealing at 52–60°C (for details see S2 Table) for 1 min and amplification at 72°C for 10 s. The analyses were carried out in StepOnePlus^™^ Real-Time PCR System (Applied Biosystems, USA).

Respective amplicons were purified by QIAquick PCR Purification Kit (Qiagen, Germany) and their lenghts were verified by DNA gel electrophoresis. From the molecular weight of the fragments and DNA concentrations measured by Quant-iT^™^ PicoGreen^™^ dsDNA Assay Kit (Invitrogen^™^, USA), the concentrations of individual amplicons (in copies per microliter) were calculated. The amplicons and the information on their concentrations were used for calibration of the different qPCR assays on the same plate (using serially diluted amplicon preparations, i.e., using standard curve method).

### Calculations and statistics

For statistical analyses of the results, we used programming language R 3.0.0 in RStudio 0.99.902 environment [58, 59]. Command "t-test" was used for Welch Two Sample t-test comparison, "lm" and "anova" for linear models and analyses of dispersion. For creating graphs, SigmaPlot (Systat Software, Inc) was used. Due to inconstant expression levels of the reference genes, the results are not normalized against the reference genes, but they are normalized against the amount of total RNA subjected to reverse transcription of each corresponding sample. Raw data are available in S3 Table.

### Analyses of plant biomass production, phosphorus acquisition, root colonization by AM fungus, and carbon fluxes in the symbiosis

In Exp 1, additional plants were harvested in regular intervals to analyse growth (biomass production), colonization of the roots by the AM fungus, plant P nutrition and C fluxes from the plant to the AM fungus (S3 Fig, S4 Fig, S5 Fig, S6 Fig, S3 Table). Plant biomass was oven-dried at 65°C to constant weight. Dried samples of root and shoot biomass were ground to powder using a ball mill (MM200, Retsch, Haan, Germany) and of that, 0.08–1 g was used for further analysis. Milled samples were incinerated in a muffle furnace at 550°C for 12 h and the resulting ashes were briefly heated to 250°C on a hot plate with 1 mL of 69% HNO_3_. The materials were then transferred to volumetric flasks through a filter paper and brought up to 50 mL with ultrapure (18 MΩ) water. Phosphorus concentration in the extracts was then measured by colorimetry at 610 nm using a Pharmacia LKB Ultrospec III spectrophotometer using the malachite green method [60]. Total phosphorus content was calculated from shoot- and root dry weight data and the concentrations of phosphorus in shoot and root biomass, respectively. Colonization of the plants by the AM fungus was measured by magnified intersection method following staining of the roots as described previously [61] and by qPCR using the mt5 marker set [62]. Carbon fluxes from the plant to the fungus and the nitrogen concentration in the plants were measured just after and 4 days after pulse-labeling the plants with ^13^CO_2_ conduced at 47 dpp, using the experimental framework described in Slavíková et al. [63].

## Results

### Mycorrhizal plants have higher P content, but do not differ in biomass from the NM plants

In Exp 1, the overall appearence (S1 Photos) of NM and M+ plants was similar in terms of the biomass production (S3 Fig). The M+ plants showed fully developed AMS, as observed by AM fungal structures including arbuscules in the M+ roots (S4 Fig), and the expression of AM-specific *PT4* transporter (see Results below). A strong difference in P concentrations between the M+ and NM plants was recorded. At 35 dpp, the M+ plants had nearly twice as high concentration of P in their tissues and at 64 dpp, the total content of P in the M+ plants was more than double of those in the NM plants (S5 Fig). Additionally to P measurements, a nitrogen content was measured, which was higher in M+ plants compared to NM plants at 47 and 51 dpp (S6 Fig). Furthermore the M+ plants assimilated more ^13^CO_2_ than NM plants, resulting in higher ^13^C content mainly in shoots at 47 and 51 dpp (S6 Fig).

### Mycorrhizal phosphate-uptake pathway is active in M+ plants and reacts to shading

**Fig 2:**
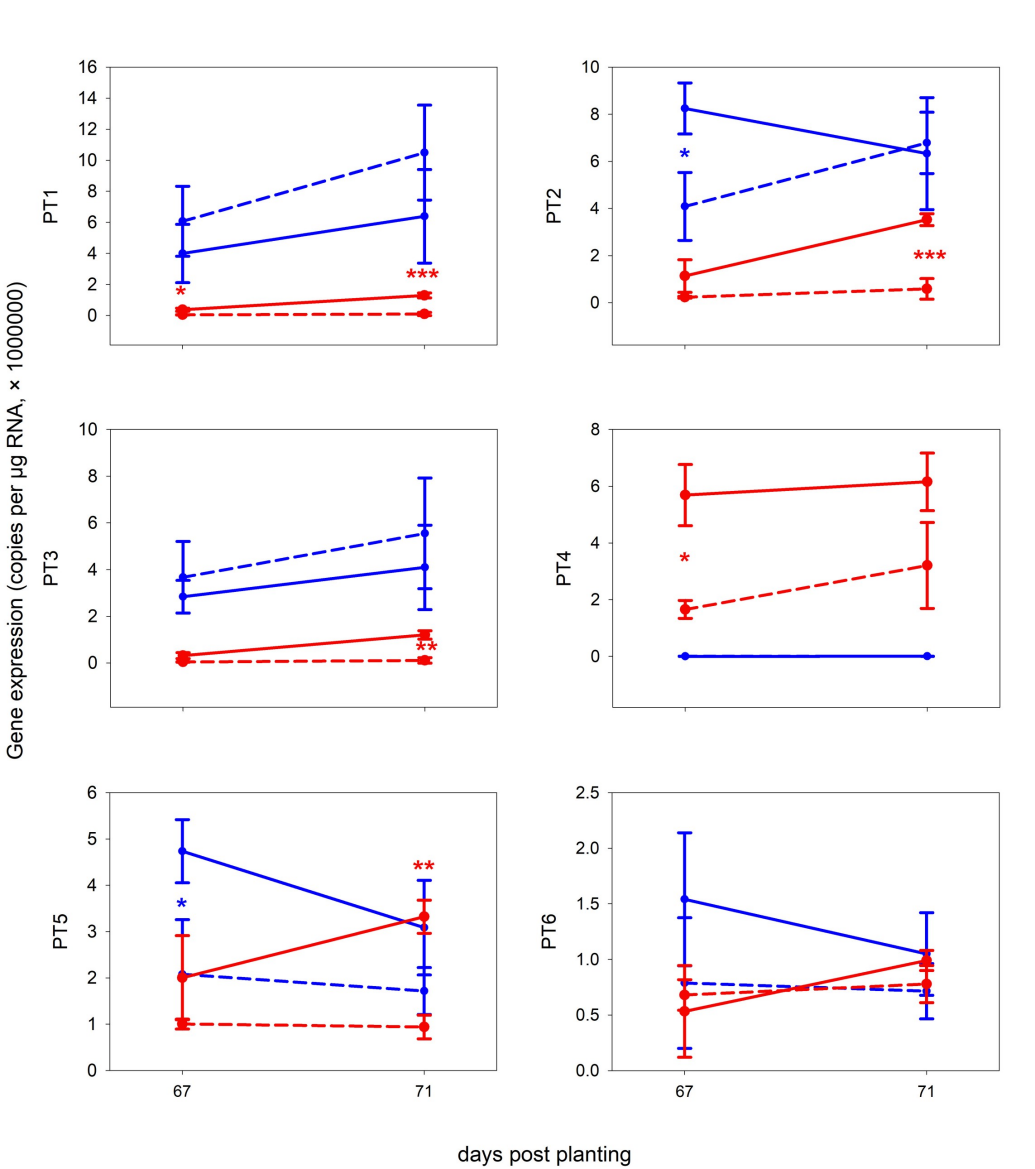
The expression of genes for high-affinity inorganic phosphate transporters (*PT1—PT6*) obtained by qPCR from roots of *M*. *truncatula* in Exp 2. X-axis: days post planting, Y-axis: number of corresponding-gene copies per 1 μg RNA subjected to reverse transcription. Red: mycorrhizal, Blue: non-mycorrhizal, Full line: full light (100%), dashed-line: shaded plants (10% of light). Error bars show standard deviations, n = 3. For further details see Materials and methods and S2 Table. Asterisks indicate significance levels (p-value classes) as per t-test comparing non-shaded and shaded plants within the mycorrhizal (red) or non-mycorrhizal (blue) treatments: 0 < *** < 0.001 ≤ ** < 0.01 ≤ * < 0.05.

The AM-specific phosphate transporter (*PT4*) was expressed specifically in the M+ roots of *M*. *truncatula* (Fig 2), while the other (direct phosphate-uptake pathway) transporters (*PT1*, *PT2* and *PT3*) were downregulated by the presence of the AM fungus. In Exp 1, this pattern was consistent in all timepoints, with exception of 63 dpp for the *PT2*. In M+ plants, the PT4 expression was about four-times higher than those of the other PTs. The expression of *PT4* was thus strongly dominating the PT-transcript-pool in M+ roots with relative proportion of 60–90% of the four PT (direct + mycorrhizal pathways). In Exp 2, short-term light deprivation only had a small effect on expression of the PTs involved in the direct P-uptake pathway, of which only *PT2* was significantly upregulated in shaded NM plants, but downregulated at other timepoints (Fig 2).

On the contrary, the mycorrhizal pathway (*PT4*) was strongly downregulated upon 90% shading in Exp 2 (Fig 2). Regarding its expression levels, PT4 was expressed about three-times lower in shaded M+ plant roots than in their unshaded M+ counterparts, but still was a dominant PT-transcript among all of the six PT homologues.

*PT5* and *PT6* played a dominant role in shoots (S7 Fig), while the proportion of their transcripts to all six PTs stably remained on the same high level (88–99%, 95% on average) in shoots, regardless of the environmental conditions. It is important to mention that *PT5* and *PT6* were significantly downregulated in M+ shoots from Exp 1 as compared to NM shoots. In roots, the ratio of PT5- + PT6-transcripts to all PT-transcripts was just around 22% on average, regardless of the other variables (harvest time or shading).

### Genes encoding saccharide transporters exhibit altered expression status in M+ roots

Using a manual-search in the whole-transcriptome data, 10 genes were selected for further analyses, which exhibited altered level of transcription upon mycorrhization. Of the available list of *M*. *truncatula*‘s saccharide transporters [54], just a few reacted on the presence of the AM fungus in the roots in our experiments—namely the probes for one tonoplast monosaccharide transporter (*TMT*) and two poyol/monosaccharide transporters (*PMT*), named here *PMTa* and *PMTb* (see S2 Table for further details).

*TMT* was significantly downregulated at two timepoints in M+ roots (S7 Fig). Unlike most of the other genes investigated here, which were almost not-expressed in shoots, *TMT* showed relatively high level of expression in the shoots, where it was upregulated at 35 dpp but downregulated at 49 and 63 dpp as compared to the NM treatment. However, in Exp 2, the downregulation of *TMT* was not significant. Upon 90% shading, M+ roots displayed significantly lower expression of *TMT* after 3 days of shading, although no change was detected in the NM roots (S7 Fig). Regarding shaded shoots, *TMT* expression was not significantly altered in the M+ shoots. In contrast, 90% shaded NM shoots upregulated the expression of *TMT* at both timepoints.

A "switch-like" character of expression was recorded for *PMTa*, which was upregulated at 35 and 49 dpp in M+ roots, but suddenly and strongly increasing the expression in the NM roots thereafter (S7 Fig). Interestingly, the expression differences were not confirmed in Exp 2 (S7 Fig). Also, the upregulation of *PMTa* in shaded roots (both M+ and NM) was not significant. In shoots, *PMTa* was only expressed to a small extent (tens of copies per 1 μg of RNA; S7 Fig).

**Fig 3:**
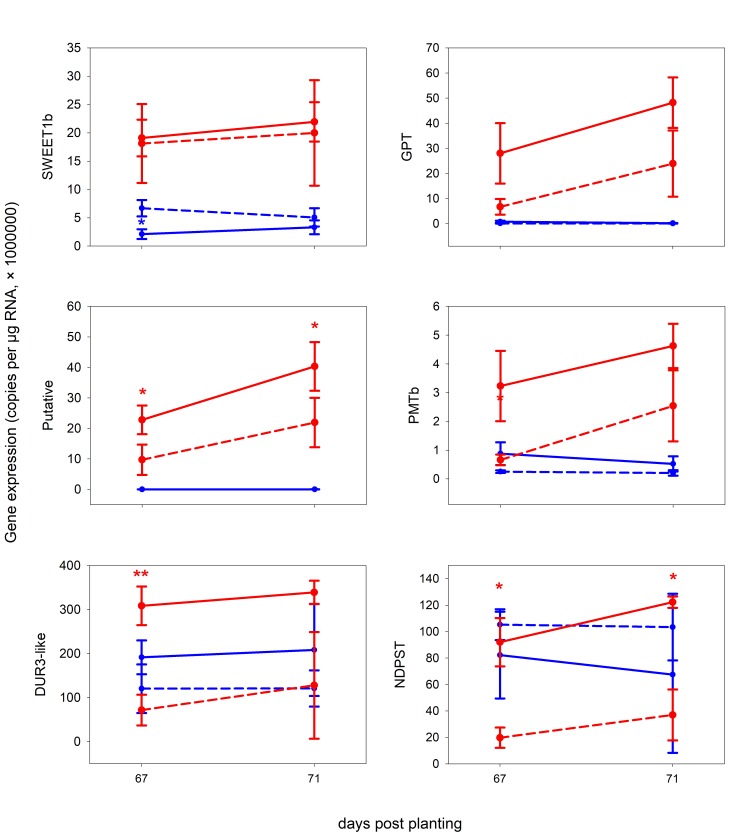
The expression of several genes obtained by qPCR from roots of *M*. *truncatula* in Exp 2. X-axis: days post planting, Y-axis: number of corresponding-gene copies per 1 μg of RNA subjected to reverse transcription. Red: mycorrhizal, Blue: non-mycorrhizal, Full line: full light (100%), dashed-line: shaded plants (10% of light). Error bars show standard deviations, n = 3. For further details see Materials and methods and S2 Table. Asterisks indicate significance levels (p-value classes) as per t-test comparing non-shaded and shaded plants within the mycorrhizal (red) or non-mycorrhizal (blue) treatments: 0 < *** < 0.001 ≤ ** < 0.01 ≤ * < 0.05.

Activation of *PMTb* in M+ roots was clearly visible (S7 Fig), however, its upregulation as compared to the NM treatment at 49 dpp was not significant. Nevertheless, Exp 2 confirmed its upregulation in M+ roots (Fig 3). Furthemore, *PMTb* was downregulated in M+ roots upon shading, with a drop to levels recorded in the NM treatment after 3 days of shading. In both M+ and NM shoots, the expression of *PMTb* was on comparable level as is in the roots. Moreover, it was upregulated once in the M+ shoots (63 dpp), and downregulated once upon 90% shading in the M+ shoots.

By searching for the keyword "sugar" using application "Samples" (Genevestigator), 113 probes with matching anotation were found, and another 3 genes encoding saccharide transpoters with altered expression in the M+ as compared to the NM treatment were selected: two bidirectional sugar transporters *SWEET1b* and *SWEET3a*, and a sugar porter *Mtst1*.

While the upregulation of *SWEET3a* in M+ roots was evident based on the microarray data (S2 Table), significant differences between the M+ and NM roots were not confirmed by the qPCR (S7 Fig). Moreover, at 63 dpp, *SWEET3a* was expressed more in the NM roots as compared to the M+ roots. Upon 90% shading in Exp 2, the expression in the M+ roots decreased, but only after 3 days of shading the results significantly differed between full light and shaded treatments (S7 Fig). Expression of *SWEET3a* in the shoots was generally lower than in the roots, it was suppressed in M+ plants as compared to the NM plants (S7 Fig) and also it was suppressed in the shoots by (longer-time) shading (S7 Fig).

The expression of *SWEET1b* appeared to be modulated by the AMS, too–its expression in NM roots was low and the upregulation in M+ roots was statisticaly significant in all timepoints included in Exp 1 (S7 Fig). Yet, the expression of the gene seems not to have reacted to shading of the plants in M+ roots, whereas in NM roots the shading upregulated its expression transiently (Fig 3). In shoots, the expression levels of this gene were close to zero (S7 Fig).

The expression of *Mtst1* [33] displayed a similar "switch-like" pattern as the *PMTa* did–it was upregulated in M+ roots at 35 dpp, then the differences disappeared and finally, at 63 dpp, *Mtst1* was downregulated (S7 Fig). In Exp 2, the expression of *Mtst1* was more or less similar to the Exp 1. In shoots, the gene was only very slighty expressed (tens of copies per 1 μg of RNA; S7 Fig).

With a similar approach, using keyword "glucose", a strong shift in expression level of glucose-6-phosphate / phosphate translocator 1 (*GPT*) gene located on chromosome 8 was found. While almost not-expressed in NM plants, we saw a clear upregulation of *GPT* in M+ roots (S7 Fig) and in reponse to short-term shading, the expression levels decreased significantly (Fig 3). The expression of this gene in shoots was close to zero (S7 Fig).

### *DUR3-like*, *NDPST* and putative transporter–novel genes in the AM symbiosis

A few other genes somewhat connected to saccharide metabolism or transportome of the plants were selected for further expression profiling, based on keywords "symport", "sugar" or "transmembrane", and their differential expression in the M+ and NM plants as assessed by the microarray analysis. Their expression level in M+ roots promised a relevance to the AMS, with direct or indirect relevance to the transfer of C from the plant to the AM fungus.

Strong upregulation of *DUR3-like* encoding gene at 35 dpp in M+ roots pointed towards a specific role of this transporter in M+ roots, though it probably symports Na^+^ and urea. The difference in expression of this gene between M+ and NM roots disappeared at 49 dpp, but it was upregulated again at 63, 67 and 71 dpp (Figs 3 and S7). Shading transiently down-regulated expression of *DUR3-like* gene in the mycorrhizal roots, yet the expression differences between shaded and non-shaded M+ plants were not significant at a longer term (Fig 3). In shoots, *DUR3-like* encoding gene was not expressed very strongly. Yet, upon shading, its expression levels dropped in both M+ and NM shoots, indicating its potential involvement in shoot metabolism regardless of the symbiosis (S7 Fig).

The only enzyme (not transporter) examined in this study was a nucleotide-diphosphosugar transferase (*NDPST*). Its expression seemed to be upregulated in M+ roots at 35 dpp, but it dropped at 49 dpp (S7 Fig) and the pattern was not the same in Exp 1 and Exp 2 (Fig 3)–however, upon shading, the expression decrease of this gene was conspicious. There was no significant difference between M+ and NM plants in the *NDPST* expression in shoots (S7 Fig).

Among poorly annotated genes, one putative membrane transporter (MTR_8g071050) was selected in this study (named *Putative* here). Similarly to *GPT* and *PMTb*, it was almost not expressed in NM roots and both M+ and NM shoots (S7 Fig), but its expression was strongly upregulated in M+ roots (S7 Fig). More importantly, upon shading, it was significantly downregulated in M+ roots (Fig 3). Based on topology prediction, using HMMTOP, CCTOP and PredictProtein, 12 transmembrane helices were predicted. It is predicted to localize into endoplasmic reticulum, but the PredictProtein do not count with PAM. Most importantly, BLASTp search in non-host speciesˈ database showed no results (see Discusion for further information).

### The expression of *GPT*, *PMTb*, *DUR3-like*, *NDPST* and *Putative* transporter correlates with expression of *PT4* in M+ roots upon shading

**Fig 4:**
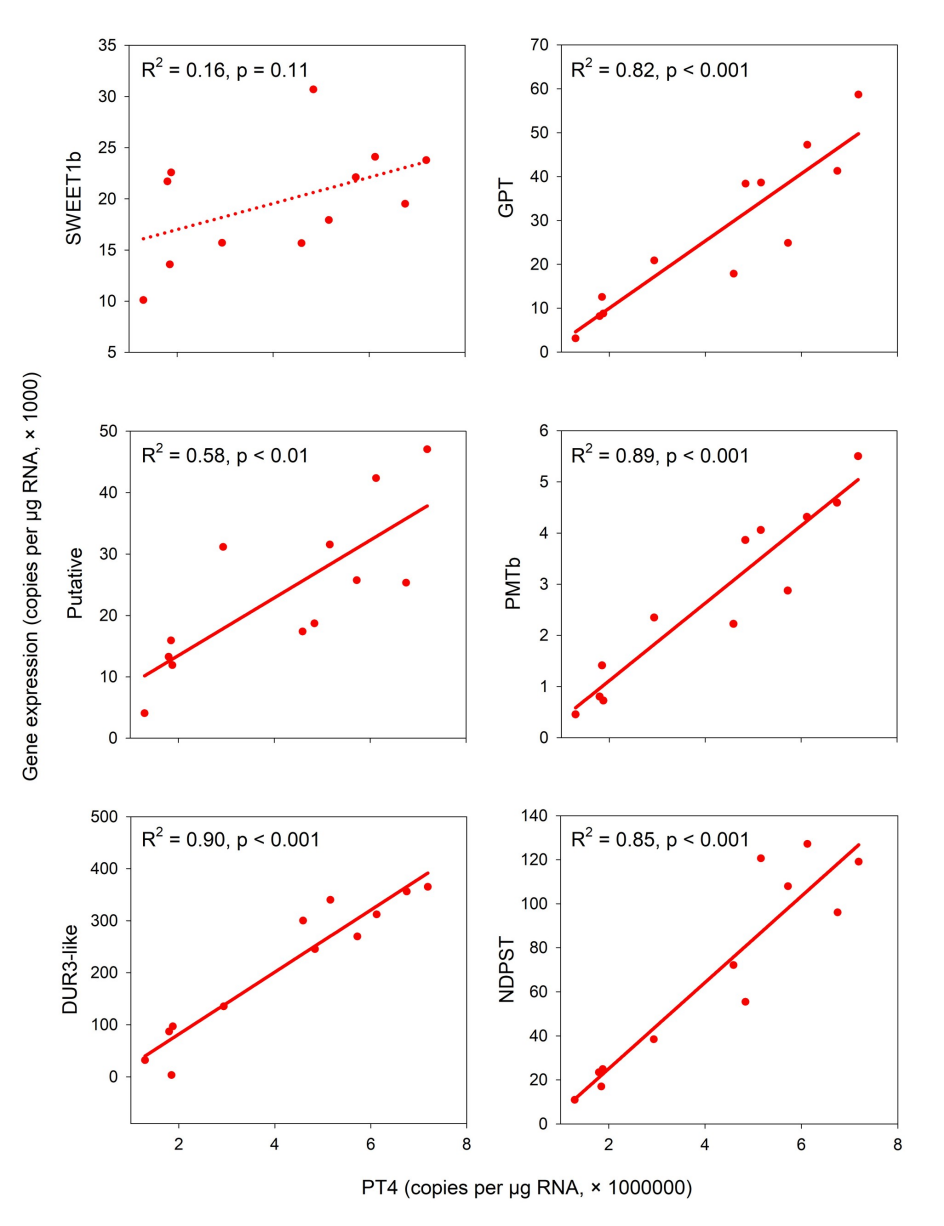
Scatterplots with simple linear regression of expressions of several genes against *PT4* expression in the mycorrhizal root samples in Exp 2. X-axis: number of *PT4* copies per 1 μg of RNA subjected to reverse transcription, Y-axis: number of corresponding-genes copies per 1 μg of RNA subjected to reverse transcription. Both harvests (67 and 71 dpp) and both light treatments (100% and 10% light) are included in the analysis. R^2^ and p-values for each specific regression are provided next to the regression lines.

In Exp 2, the expression levels of PT4 decreased significantly in response to the shading of the plants. We used this induced response to correlate expressional changes of the other genes tested in this study in M+ roots with those of *PT4*. Several genes displayed very similar pattern of downregulation upon shading as the *PT4* gene (Fig 4), while some other genes showed no correlation. For shoot samples, no correlations were evaluated.

The *SWEET1b* gene did not change its expression in response to shading, which was further supported by *p* value greater than 0.11 for simple linear correlation with *PT4* expression in the M+ roots. On the contrary, the simple linear correlations of *GPT*, *PMTb*, *DUR3-like*, *NDPST* and *Putative* transporter expressions with the expression of *PT4* were all significant (Fig 4). Since the differences in expression of those genes in roots of M+ shaded and non-shaded plants were mostly significant (Fig 3), we assume a direct involvement of these genes in AMS, at least regarding their transcriptional regulation.

In terms of the expression patterns of the above genes, there were three distinct patterns (Fig 3). For *GPT1* and *Putative* transporter genes the situation was identical as for the *PT4*: In NM plants, the genes were virtually not expressed, but in M+ plants, they were expressed and downregulated by shading (Fig 4). For *PMTb* and *DUR3-like* genes, the situation with respect to response to shading in M+ plants was analogous to *GPT1* and *Putative*: the genes were significantly downregulated upon shading in M+ plants and the correlation with *PT4* was significant, too. However, in contrast to *GPT1* and *Putative* transporter, *PMTb* and *DUR3-like* genes were also expressed in the NM plants, yet in a generally lower level than in the M+ plants. Finally, *NDPST* gene was expressed similarly in both NM and M+ plants exposed to full light, but upon shading, it was (significantly) downregulated only in M+ roots (Fig 3) and well correlated with *PT4* expression (Fig 4).

## Discussion

In this study we grew barrel medic (*M*. *truncatula*) with compatible rhizobia and with or without AM fungus *R*. *irregularis*. This common AMS model system was used in two separate experiments, Exp 1 and Exp 2, to identify novel genes possibly involved in shuffling reduced C compounds from the plant to the AM fungus (Fig 1), and to assess response in expression of these genes upon short-term light deprivation according to previously described experimental setup [64], respectively. AMS was fully established in the M+ plants, as shown by intensive AM fungal development in the roots in Exp 1 (S4 Fig), as well as by the expression of AM-specific *PT4* gene (Fig 2). The NM plants remained free from the AM fungal colonization. Interestingly, the plants of both treatments were similar in terms of biomass production (S3 Fig), whereas the M+ plants had twice as high P concentration as the NM plants (S5 Fig). This indicates a functional mycorrhizal P uptake pathway, and because of the lack of positive growth response of the plants to AMS formation, it also indicates an extensive volume of trade of P for C between the symbionts [65, 66], presumably at the periarbuscular interface.

**Fig 5:**
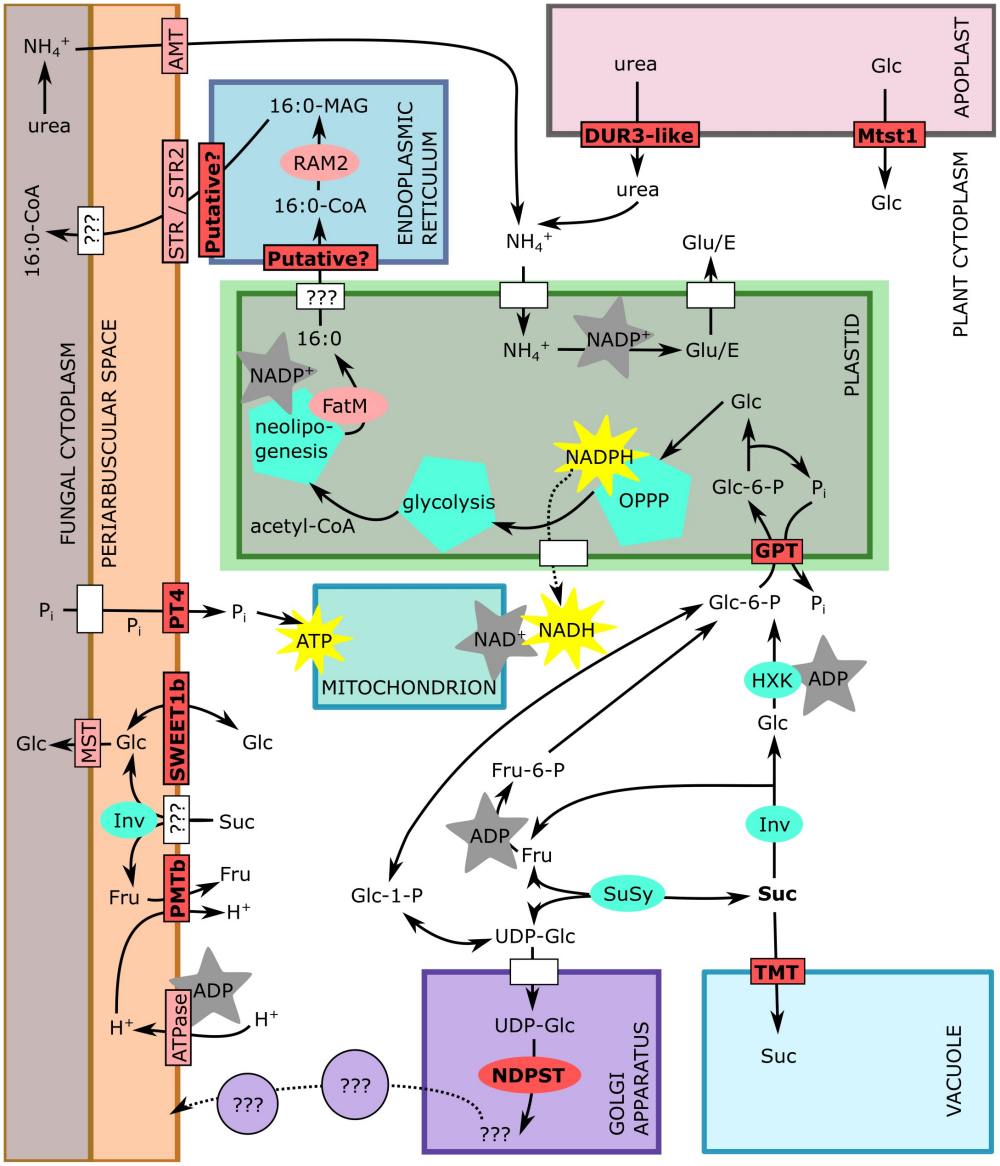
Scheme of arbuscocyte showing genes specifically studied in our research (bright red), and some other of AM symbiosis-relevant proteins (light red), enzymes and enzymatic pathways (cyan), and not specified proteins (white). Generation of energy molecules (ATP) and reductive coenzymes (NADH / NADPH) is indicated by yellow stars, while consuming those compounds is indicated by gray stars. 16:0, free palmitoyl; 16:0-CoA, palmitoyl-Coenzyme A; 16:0-MAG, mono acyl glycerol (palmitoyl glycerol); acetyl-CoA, acetyl-Coenzyme A; UDP-Glc, uridine diphosphate glucose; Glc-1-P, glucose-1-phosphate; Glc-6-P, glucose-6-phosphate; Fru-6-P, fructose-6-phosphate; P_i_, phosphate; Inv, invertase; SuSy, sucrose synthase; Suc, sucrose; Glc, glucose; Fru, fructose; HXK, hexokinase; OPPP, oxidative pentose phosphate pathway; Glu/E, glutamate.

Among genes encoding saccharide transporters, we have identified transcriptional changes of two SWEET genes of *M*. *truncatula*–*SWEET1b* and *SWEET3a*. SWEET proteins are pH-independent, bidirectional ST with broad representation in bacteria, plants, fungi and animals [67–69] with crucial roles in multiple biological processes. *SWEET1b* shows upregulation in M+ plants, similar to the rice gene *OsSWEET1b* [70] or potato genes *StSWEET1a* and *StSWEET1b* [71]. Closest characterized homologue is *AtSWEET1* of thale cress (*Arabidopsis thaliana* (L.) Heynh), whose substrate specificity is high for Glc and low for galactose, but does not transfer Suc [67]. We assumed a similar substrate specificity for the *SWEET1b*, which was also experimentally confirmed recently by An et al. [34]. Usually, the homologues of this gene are expressed in plant sink tissues like fruit, nodules, tubers or upon mycorrhization, so the role in apoplastic transport of Glc is assumed. Notably, *SWEET1b* localizes on PAM [34], so it allows Glc to leak out, where *RiMST* of the *R*. *irregularis* probably takes up the Glc molecules immediately, and simultaneously, it serves as passive retractor of Glc to the plant cells, with following utilisation of Glc in plant cytoplasm or sequestring it into a vacuole (Fig 5). The fungal ST posses high-affinity to Glc–*RiMST2* has a K_m_ of 33 ± 12.5 μM [35], and *AtSWEET1* is a low-affinity Glc transporter with a K_m_ of 9 mM [67]. That means, the net efflux of Glc from arbuscocyte is expected. On the other hand, the Glc retracted from periarbuscular space by *SWEET1b* will be actively utilised by cytoplasmic hexokinase to Glc-6-P, and since the hexokinase has a K_m_ of 20–130 μM for Glc [72], the competition for Glc between plant and fungus may occur even when no active ST is located at the PAM (Fig 5). Based on expression profiling of *SWEET3a* in our study, it seems that expression of this particular gene is not absolutely required for the AMS functioning, although it is affected by the symbiosis in some situations (see S7 Fig for more information).

Another AM-specific candidate gene is *GPT*. This gene has longer mRNA (contrary to the other 2 GPT genes in *M*. *truncatula* genome) and is annotated also as O-glycosyl hydrolase. Since no substrate specificity has been establisled for this gene with respect to hydrolysis, this enzymatic annotation still needs to be further investigated. *GPT* in general imports Glc-6-P to plastids in exchange of P to maintain starch synthesis, oxidative pentose-phosphate pathway (OPPP), neolipogenesis or nitrite reduction, mainly in heterotrophic tissues [73]. This crossroad of cell metabolism secures C flow to plastid, while enabling sequestration towards storage (anabolism) or catabolism and symbiont-feeding by lipids (Fig 5). In plastids, products of OPPP may serve to feed the shikimate pathway producing aromates, the methylerithritolphosphate pathway (MEPP) producing isoprenoids, for synthesis of nucleotides, and other molecules like phytohormones [74]. Assuming that all these metabolic pathways are already active in the cells of NM plants, this *GPT* can hardly be the main C importer of plastids, since we measured almost zero expression in the NM plant roots (Figs 3 and S7). On the other hand, we show that *GPT* is an AM-specific gene, which is regulated in the same manner as *PT4*. It would be interesting to further investigate if *GPT* expression and / or activity is directly linked to presumed lipid-transfer machinery like *STR*/*STR2* [44, 75].

According to the gene expression atlas (MtGEA; https://mtgea.noble.org/v3/), *PMTb* is expressed in nodules, and there is no expression pattern recorded as yet of *PMTb* in the AMS. On the contrary, nodules were present in all treatments, although nodulation was not specifically quantified in our experiments, thus quantitative differences may have escaped our attention. Closest *A*. *thaliana* homologues symports Fru and xylitol with H^+^ so it activelly imports Fru from apoplast after cleavage of Suc [76]. Even a small change in *PMTb* activity on PAM should be sufficient to actively resorb Fru, because even a small amount of transporters may effectively change the concentrations of solutes in the periarbuscular space [77]. However, the precise localization and function of this gene is remaining an unsolved puzzle.

The homologue of *DUR3-like* gene in *Zea mays*, Zm*DUR3*, is a symporter of urea and sodium ions [78] and it was described to mediate the retrieval of urea from senescensing *Arabidopsis* leaves‘ apoplast [79]. The periarbuscular space is a type of apoplastic compartment, so the urea could be taken up by *DUR3-like*, but since the exchange of NH_4_^+^ between the plant and the fungus is fairly described [80–82], this option is unlikely. More likely, the urea derives from senescent structures like root cells or degenerated arbuscules, since the arbuscules are ephemeral structures [83].

*NDPST* glycosylates its substrates and this one *NDPST* (out of 23 described *NDPST M*. *truncatula* genes) is annotated as glucosylating by UDP-Glc. Does it posses a role in glucosylation of membrane lipids or proteins? The best possibility appears to be an involvement in cell wall component synthesis in Golgi apparatus (Fig 5)–the cell wall biogenesis [84]. But since there is no degradation of plant cell wall polymers by AM fungi [85, 86], there is probably no reason for delivering cell wall polymers into periarbuscular space. In this study, we have discovered a strong evidence for AM-dependent regulation of *NDPST*, which displays a similar pattern as the regulation of *PT4*, i.e., significantly responding to a short-term shading of the plants. Another *NDPST* (MTR_8g069400) is predicted to be AM-conserved gene [41], so the relevance of (a change of) glycosylation upon AMS appears as a relevant possibility. Interestingly, the lipid pathway was depicted as a variation of cutin biosynthesis pathway [38], and higher production of cell wall components is a common defence strategy against fungal pathogens [87, 88].

*Putative* membrane transporter showed a very interesting expression pattern in our experiments. Because it was predicted to posses 12 transmembrane helices, which is a common feature of proteins from Major Facilitator Superfamily [89], it may well be involved in membrane-bound exchanges in AMS. We present three arguments here for possible involvement of this *Putative* membrane transporter in the AMS. First, we found almost zero expression in NM roots, indicating that it is an AM-induced gene (Figs 3 and S7). Second, its downregulation in shaded M+ roots is significantly correlated with the downregulation of *PT4*, so it appears as AM-trade controled gene (Fig 4). Third, simple BLASTp search (https://blast.ncbi.nlm.nih.gov/Blast.cgi) of its protein sequence (XP_013446273.1) results in hits in broad range of land plant families, but no hit can be found when narrowing the query to *Amaranthaceae* or *Brassicaceae* family only, which are known NM families [90]. Recently, it has been shown that phylogenomic approach is useful for identification of AMS-conserved genes, when the orthologues are present in AM-species and absent in NM-species [41]. Therefore, we assume that this uncharacterized *Putative* membrane transporter has a specific function in the AMS and thus deserves a particular attention in future research.

Based on differential gene expression, we have identified several novel genes in AMS research and we propose their involvement in symbiotic trade in AMS. Their genuine involvement in AMS needs to be verified in future research. Such quest is not easy, because same physiological reactions, like sugar partitioning among tissues and plant organs, can be facilitated by different molecular mechanisms in distantly related plant species [22]. The properties of ST, like substrate specificity, transport activity or localization may differ markedly, whereas sequence comparisons may still show strong similarities. Also, the subcellular localization of the ST can change in the same plant [91]. The next step for deciphering sugar partitioning in AM plants is the biochemical characterization and subcellular localization of plant ST during AMS at protein level mainly. There definitely is still much to be done with respect to full undestanding of the molecular mechanisms of bidirectional trading of resources in the AMS, particularly in respect of C flux from plants to the AM fungus, including different C forms [92]. Yet this appears necessary to achieve the next break-through in understanding of the functioning of this ancient interkingdom relationship [5] and possibly also in its better utilization for human welfare [93, 94].

## Supporting information

S1 Table Light intensity and temperature in the experimental glasshouse. Raw data of light intensity (lux) and temperature (°C) continuously measured every 15 min in the experimental glasshouse during growth of the plants by datalogger Testo 435–2 equipped with both lux and temperature probes. List 1: Environmental conditions during Exp 1; List 2: Environmental conditions during Exp 2. The sensors were moved from full light to the shading tent and back several times during the experiment–the points of transition are noted down.
(XLSX)

S2 Table Summary of investigated genes. A table of genes specifically focused on in our research with their abbreviations used throughout the text, the gene symbol used in 4^th^ version of *M*. *truncatula* genome annotation, available annotations of the genes, qPCR parameters (annealing temperatures and the sequences of primers used), the source of gene discovery and probe set IDs. Lower part: A screenshot of expression potential of investigated genes from Genevestigator software.
(XLSX)

S3 Table Raw data from the qPCR analyses, plant biomass, phosphorus and nitrogen nutrition, root colonization and carbon flux measurements. This table contains raw data from which the graphs presented in the paper were made. List 1: The qPCR results showing detected numbers of corresponding mRNA copies per 1 μg of RNA subjected to reverse transcription; List 2: Dry mass of the plants, phosphorus concentrations and content in the plants and colonization of the roots; List 3: Carbon isotopic composition in the different plant tissues and the ^13^C allocation into the different system compartments (shoots, roots, substrate, belowground respired CO_2_), and the nitrogen concentration in the plants.
(XLS)

S1 Fig Agarose gel electrophoresis of the RNA used for microarray-based gene expression analyses. Materials & methods and results of total RNA quality control by agarose gel electrophoresis of RNA samples from 35 dpp (Exp 1).
(DOC)

S2 Fig RNA quality control by Agilent 2100 Bioanalyser before microarray-based gene expression analyses. Original data sheet showing results of RNA quality control processed by Agilent 2100 Bioanalyser prior to Affymetrix microarray analysis. Total RNA deprived of DNA contaminations from 35 dpp (Exp 1) was used. Samples 1–6 (4S-9S) correspond to the shoot samples and 7–12 (4R-9R) to the root samples, while samples 1–3 & 7–9 (4S-6S & 4R-6R) correspond to mycorrhizal treatment and samples 4–6 & 10–12 (7S-9S & 7R-9R) to non-mycorrhizal treatment.
(PDF)

S3 Fig Dry mass of *Medicago truncatula* plants in Exp 1. (A) total dry mass of whole plants per pot; (B) root to shoot ratio; (C) dry mass of whole shoots per pot; (D) dry mass of whole roots per pot. Error bars show standard deviations, n = 3. Red: mycorrhizal, Blue: non-mycorrhizal plants. Asterisks indicate significance levels as per t-test comparing mycorrhizal and non-mycorrhizal treatments at the different timepoints: 0.01 ≤ * < 0.05. When no asterisk is displayed, the values did not significantly differ between the mycorrhizal and non-mycorrhizal treatments (i.e., p ≥ 0.05).
(TIF)

S4 Fig Root colonization by the mycorrhizal fungus of the mycorrhizal plants in Exp 1. Colonization of the plant roots measured by magnified intersection method following staining of the roots (left panel) as described previously [61], with the solid line and black color indicating root occupancy by the fungal hyphae, dashed line and dark gray color standing for arbuscules, and dotted line and light gray color standing for vesicles (non-mycorrhizal root samples did not show any colonization and the results are thus not displayed), and by quantitative real-time PCR (right panel) using the mt5 marker set [62]. Red: mycorrhizal, Blue: non-mycorrhizal plants. Mean values of 3 replicate values per each timepoint are shown, error bars indicate ± standard deviations.
(TIF)

S5 Fig Phosphorus content of the plants in Exp 1. Total phosphorus content in whole plants per pot (upper panel), phosphorus concentration in the shoots (middle panel) and phosphorus concentration in the roots (lower panel). Error bars show standard deviation, n = 3. Red: mycorrhizal, Blue: non-mycorrhizal plants. Asterisks indicate significance levels as per t-test comparing mycorrhizal and non-mycorrhizal treatments at the different timepoints: 0 < *** < 0.001 ≤ ** < 0.01 ≤ * < 0.05. When no asterisk is displayed, the values did not significantly differ between the mycorrhizal and non-mycorrhizal treatments (i.e., p ≥ 0.05).
(TIF)

S6 Fig Nitrogen content of the plants and the δ^13^C values in their shoots and roots, and the ^13^C excess partitioning in Exp 1. Carbon isotopic composition (right panels) and nitrogen concentrations (left panels) measured just after (within 15 min) and 4 days after pulse-labeling the plants with ^13^CO_2_ at 47 dpp, using the experimental framework described in Slavíková et al. [63]. Error bars show standard deviations, n = 7. Asterisks indicate significance levels as per t-test comparing mycorrhizal (red or M+) and non-mycorrhizal (blue or NM) treatments at the different timepoints, or between the groups: 0 < *** < 0.001 ≤ ** < 0.01 ≤ * < 0.05. When no asterisk is displayed, the values did not significantly differ between the mycorrhizal and non-mycorrhizal treatments or the other sample groups (i.e., p ≥ 0.05).
(TIF)

S7 Fig **The expression of genes measured by the quantitative real-time PCR in *M*. *truncatula* roots (A and C) or shoots (B and D) in Exp 1 (A and B) or Exp 2 (C and D), respectively.** X-axis: days post planting, Y-axis: number of corresponding-gene transcript copies measured per 1 μg of RNA subjected to reverse transcription. Red: mycorrhizal treatment, Blue: non-mycorrhizal treatment; Full line: full light (100%), dashed-line: shaded plants (10% of light). Error bars show standard deviations, n = 6 or 3 for Exp 1 or Exp 2, respectively. For further details see Materials and methods and S2 & S3 Tables. Asterisks indicate significance levels as per t-test comparing mycorrhizal (red) and non-mycorrhizal (blue) treatments (Exp 1) or non-shaded and shaded plants within the mycorrhizal or non-mycorrhizal treatments (Exp 2) at the different timepoints: 0 < *** < 0.001 ≤ ** < 0.01 ≤ * < 0.05. When no asterisk is displayed, the values did not significantly differ between the mycorrhizal and non-mycorrhizal treatments (i.e., p ≥ 0.05).
(PDF)

S1 Photos Photos of *Medicago truncatula* plants. Photo 1: *M*. *truncatula* plants harvested from one pot at 45 dpp (Exp 1)–examples of whole root and whole shoot samples. Photo 2: Non-shaded *M*. *truncatula* plants in pot cultures during growth at 64 dpp (Exp 2). Photo 3: Shaded *M*. *truncatula* plants in pot cultures during growth at 64 dpp (Exp 2).
(PDF)

## Abbreviations

qPCR: quantitative polymerase chain reaction
PT: phosphate transporter
ST: saccharide transporter
GPT: glucose-6-phosphate/phosphate translocator
G6P: glucose-6-phosphate
Mt: *Medicago truncatula*
AM: arbuscular mycorrhizal
C: carbon
N: nitrogen
P: phosphate
M+: mycorrhizal treatment
NM: non-mycorrhizal treatment
RPM: rotations per minute
DEPC: diethylpyrocarbonate
SDS: sodiumdodecylsulphate
EDTA: ethylenediaminetetraacetic acid
EtOH: ethanol
Rnase: ribonuclease
Dnase: deoxyribonuclease
RNA: ribonucleic acid
DNA: deoxyribonucleic acid
cDNA: complementary DNA
Suc: sucrose
Glc: glucose
Fru: fructose
PAM: periarbuscular membrane
Dpp: days post planting
RT: reverse transcription
AMS: arbuscular mycorrhizal symbiosis
