# Supplementary material for: Correlative evidence for co-regulation of phosphorus and carbon exchanges with symbiotic fungus in the arbuscular mycorrhizal Medicago truncatula
Source: PLoS One. 2019 Nov 11;14(11):e0224938. doi: 10.1371/journal.pone.0224938 (PMC6844471; doi:10.1371/journal.pone.0224938)
Supplement: S1 Fig — Materials & methods and results of total RNA quality control by agarose gel electrophoresis of RNA samples from 35 dpp (Exp 1). (DOC) [file pone.0224938.s004.doc]

# Figure S1 – Agarose gel electrophoresis of RNA (Exp 1, 35 dpp)

To check the RNA quality prior to microarray analysis, we made a agarose gel electrophoresis of denaturated RNA samples. Whole shoot and whole root total RNA of 6 mycorrhizal (M+) and 6 non-mycorrhizal (NM) pots harvested at 35 dpp (Exp 1) was subjected to denaturation and checked on a denaturing agarose gel. From those, a half of samples was used for microarray analysis afterwards.

# Materials and methods

The total RNA samples were denaturated: 2 µl of samples was mixed with 2 µl of 2X RNA Gel Loading Dye (Thermo Fisher Scientific, USA) and heated to 70°C for 2 min and than rapidly chilled on ice. From those, 2.5 µl of dyed denaturated samples were loaded on gel.

The gel contained 2% of agarose (Top-Bio, Czech Republic) in TAE buffer (Sigma-Aldrich, USA). Two ladders were used: 1.5 µl of 0.5-10 Kb RNA Ladder (Ambion, USA) and 1 µl of Fast DNA Ladder (NEB, USA) per well. The electrophoresis took 40 min at 80V.

Results

## (A) Agarose gel electrophoresis of RNA of root samples

From the left: Fast DNA Ladder, 0.5-10 Kb RNA Ladder (not denaturated), 0.5-10 Kb RNA Ladder (denaturated), 6 samples of M+ roots, 6 samples of NM roots, Fast DNA Ladder

## (B) Agarose gel electrophoresis of RNA of shoot samples

From the left: Fast DNA Ladder, 0.5-10 Kb RNA Ladder (not denaturated), 0.5-10 Kb RNA Ladder (denaturated), 6 samples of M+ shoots, 6 samples of NM shoots, Fast DNA Ladder


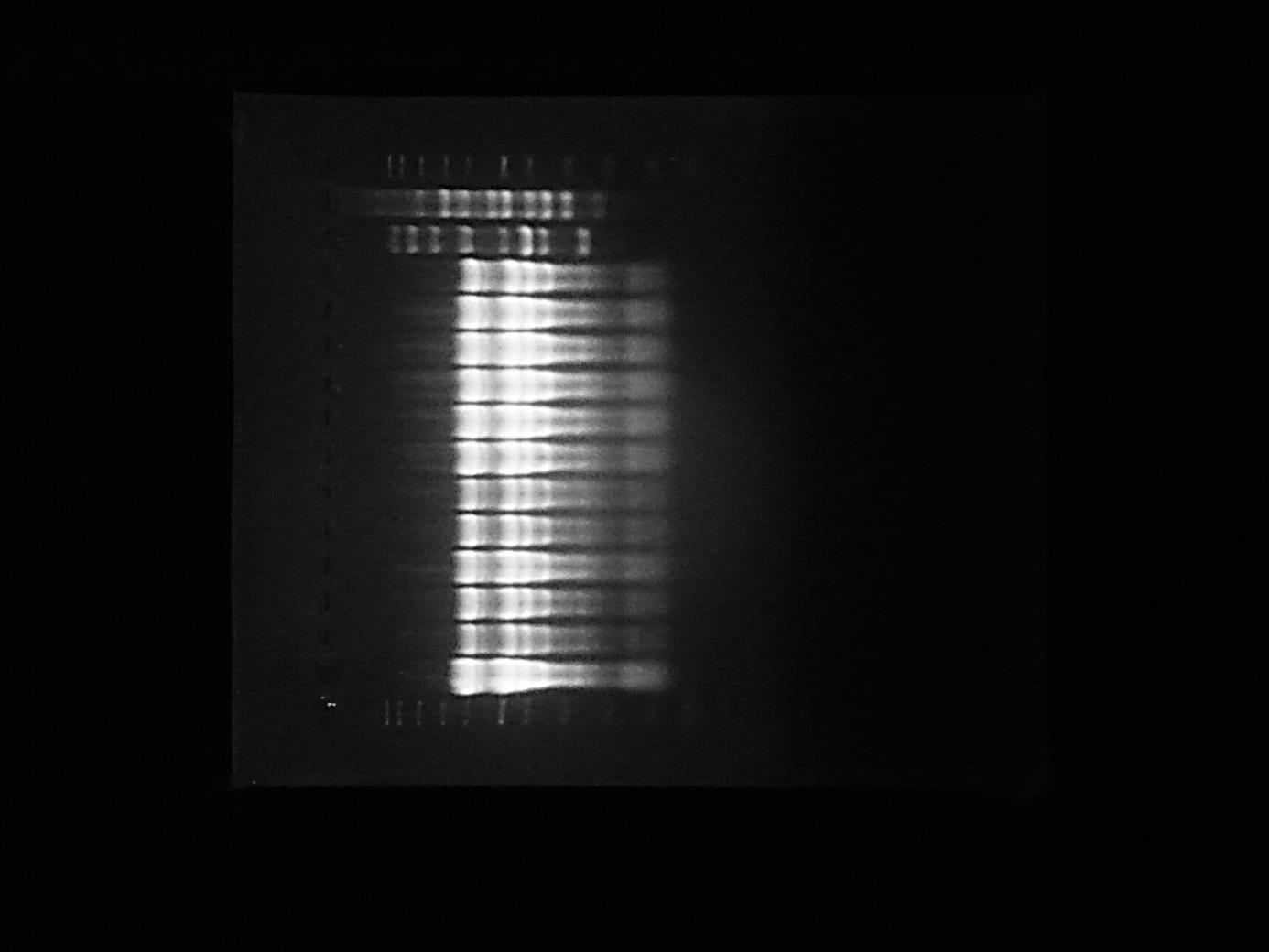


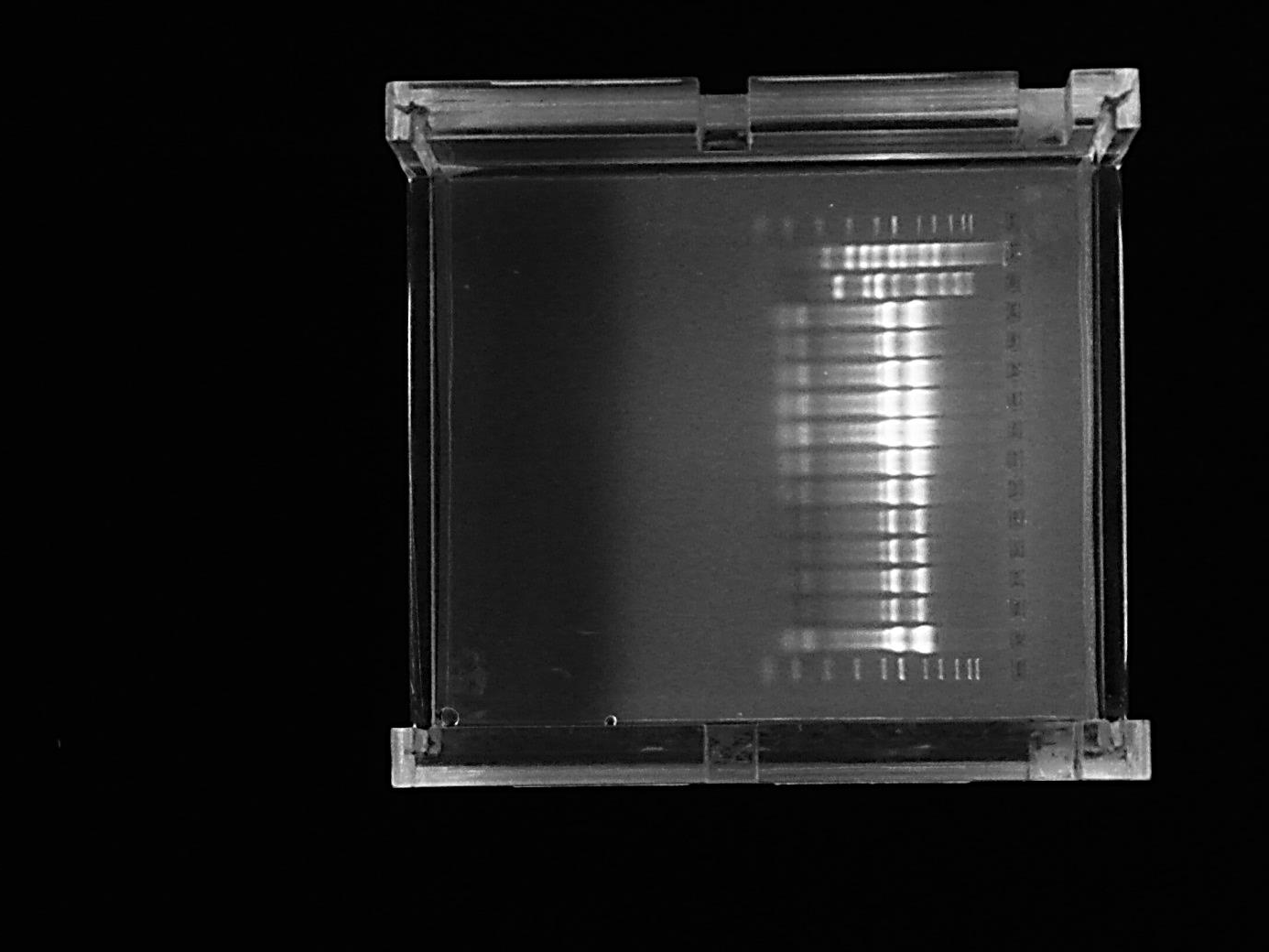


**A**

**A**

**B**
