# Supplementary material for: Correlative evidence for co-regulation of phosphorus and carbon exchanges with symbiotic fungus in the arbuscular mycorrhizal Medicago truncatula
Source: PLoS One. 2019 Nov 11;14(11):e0224938. doi: 10.1371/journal.pone.0224938 (PMC6844471; doi:10.1371/journal.pone.0224938)
Supplement: S2 Fig — Original data sheet showing results of RNA quality control processed by Agilent 2100 Bioanalyser prior to Affymetrix microarray analysis. Total RNA deprived of DNA contaminations from 35 dpp (Exp 1) was used. Samples 1–6 (4S-9S) correspond to the shoot samples and 7–12 (4R-9R) to the root samples, while samples 1–3 & 7–9 (4S-6S & 4R-6R) correspond to mycorrhizal treatment and samples 4–6 & 10–12 (7S-9S & 7R-9R) to non-mycorrhizal treatment. (PDF) [file pone.0224938.s005.pdf]

Assay Class: Plant RNA Nano
Data Path: D:\...00 expert_Plant RNA Nano_DE34904209_2015-04-22_12-16-22.xad

Created: 4/22/2015 12:16:22 PM
Modified: 4/22/2015 12:40:15 PM

Electrophoresis File Run Summary

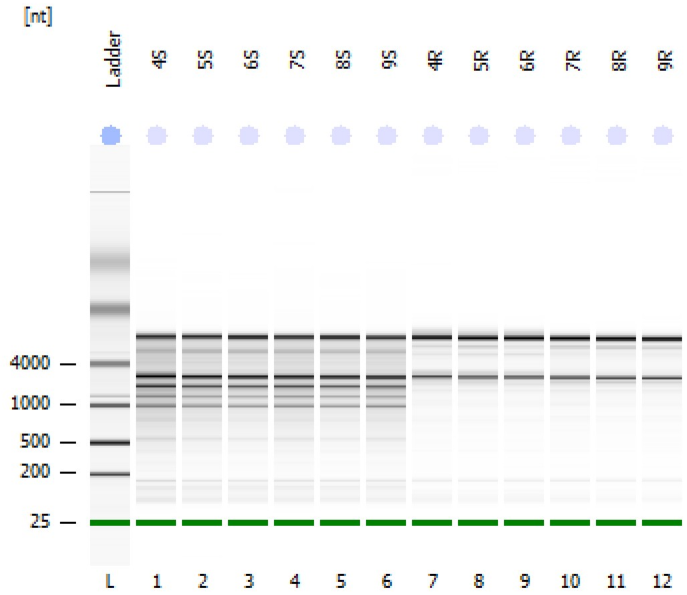

Instrument Information:

Instrument Name:	IMG-CORE-AGILENT	Firmware:	C.01.069
Serial#:	DE34904209	Type:	G2938C

Assay Information:

Assay Origin Path:	C:\Program Files\Agilent\2100 bioanalyzer\2100 expert\assays\RNA\Plant RNA Nano.xsy
Assay Class:	Plant RNA Nano
Version:	1.3
Assay Comments:	Total RNA Analysis ng sensitivity (Plant)

© Copyright 2003 - 2009 Agilent Technologies, Inc.

Chip Information:

Chip Lot #:

Reagent Kit Lot #:

Chip Comments:

4S
Jansa_MBU, RNA
RIN: 5.90

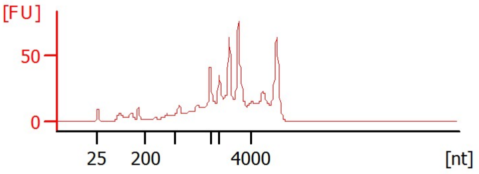

5S
Jansa_MBU, RNA
RIN: 6.10

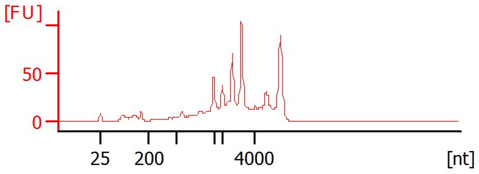

6S
Jansa_MBU, RNA
RIN: 6.40

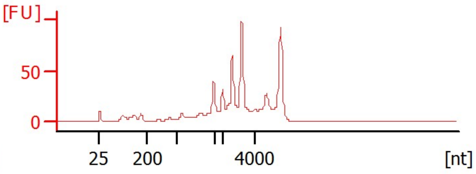

7S
Jansa_MBU, RNA
RIN: 6.10

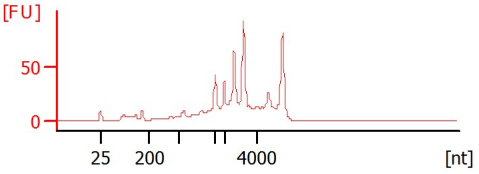

8S
Jansa_MBU, RNA
RIN: 6.40

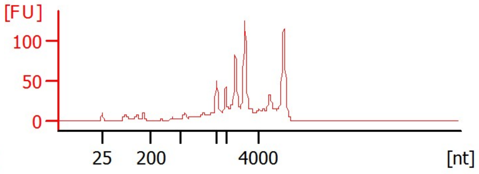

9S
Jansa_MBU, RNA
RIN: 6.10

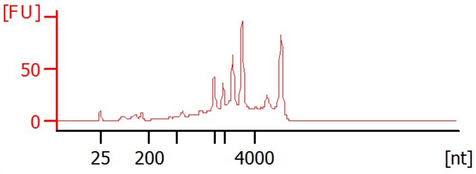

4R
Jansa_MBU, RNA
RIN:9

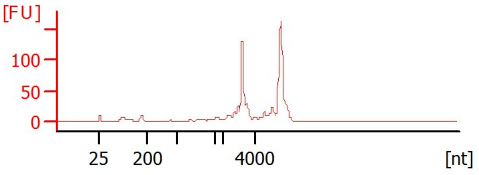

5R
Jansa_MBU, RNA
RIN: 9.20

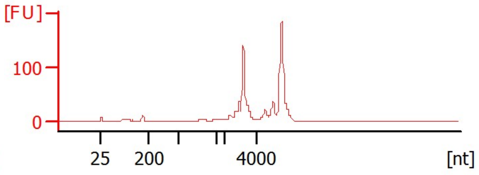

6R
Jansa_MBU, RNA
RIN: 9.40

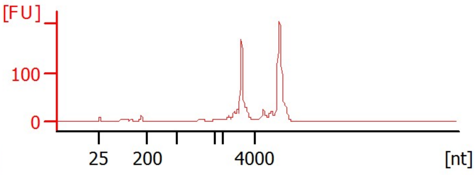

7R
Jansa_MBU, RNA
RIN: 8.90

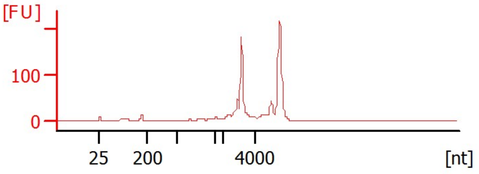

8R
Jansa_MBU, RNA
RIN: 9.30

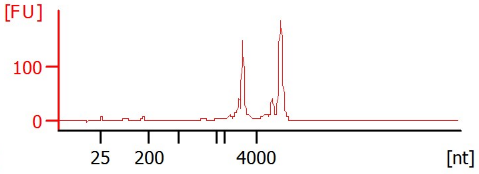

9R
Jansa_MBU, RNA
RIN: 9.20

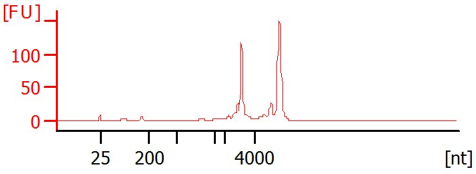

Assay Class: Plant RNA Nano
Data Path: D:\...00 expert_Plant RNA Nano_DE34904209_2015-04-22_12-16-22.xad
Created: 4/22/2015 12:16:22 PM
Modified: 4/22/2015 12:40:15 PM

Electrophoresis File Run Summary (Chip Summary)

Sample Name	Sample Comment	Status	Result Label	Result Color
4S	Jansa_MBU, RNA	✔	RIN: 5.90	
5S	Jansa_MBU, RNA	✔	RIN: 6.10	
6S	Jansa_MBU, RNA	✔	RIN: 6.40	
7S	Jansa_MBU, RNA	✔	RIN: 6.10	
8S	Jansa_MBU, RNA	✔	RIN: 6.40	
9S	Jansa_MBU, RNA	✔	RIN: 6.10	
4R	Jansa_MBU, RNA	✔	RIN:9	
5R	Jansa_MBU, RNA	✔	RIN: 9.20	
6R	Jansa_MBU, RNA	✔	RIN: 9.40	
7R	Jansa_MBU, RNA	✔	RIN: 8.90	
8R	Jansa_MBU, RNA	✔	RIN: 9.30	
9R	Jansa_MBU, RNA	✔	RIN: 9.20	
Ladder		✔	All Other Samples	

Chip Lot # **Reagent Kit Lot #**

Chip Comments :

© Copyright 2003 - 2009 Agilent Technologies, Inc.

Assay Class: Plant RNA Nano
Data Path: D:\...00 expert_Plant RNA Nano_DE34904209_2015-04-22_12-16-22.xad
Created: 4/22/2015 12:16:22 PM
Modified: 4/22/2015 12:40:15 PM

Electrophoresis Assay Details

General Analysis Settings

Number of Available Sample and Ladder Wells (Max.) : 13
Minimum Visible Range [s] : 17
Maximum Visible Range [s] : 70
Start Analysis Time Range [s] : 19
End Analysis Time Range [s] : 69
Ladder Concentration [ng/µl] : 150
Lower Marker Concentration [ng/µl] : 0
Upper Marker Concentration [ng/µl] : 0
Used Lower Marker for Quantitation
Standard Curve Fit is Logarithmic
Show Data Aligned to Lower Marker

Integrator Settings

Integration Start Time [s] : 19
Integration End Time [s] : 69
Slope Threshold : 0.6
Height Threshold [FU] : 0.2
Area Threshold : 0.1
Width Threshold [s] : 0.2
Baseline Plateau [s] : 6

Filter Settings

Filter Width [s] : 0.5
Polynomial Order : 4

Ladder

Ladder Peak	Size
1	25
2	200
3	500
4	1000
5	2000
6	4000

© Copyright 2003 - 2009 Agilent Technologies, Inc.

Assay Class: Plant RNA Nano
Data Path: D:\...00 expert_Plant RNA Nano_DE34904209_2015-04-22_12-16-22.xad

Created: 4/22/2015 12:16:22 PM
Modified: 4/22/2015 12:40:15 PM

Electropherogram Summary

Ladder

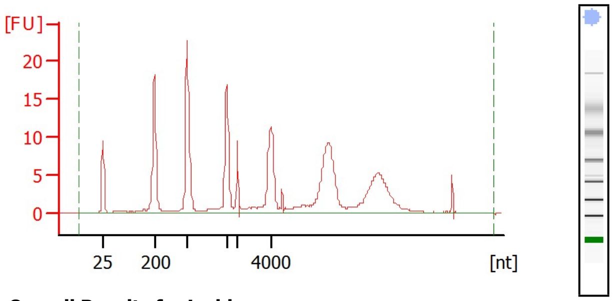

Overall Results for Ladder

RNA Area:	212.7
RNA Concentration:	150 ng/µl
Result Flagging Color:	
Result Flagging Label:	All Other Samples

4S [Jansa_MBU, RNA]

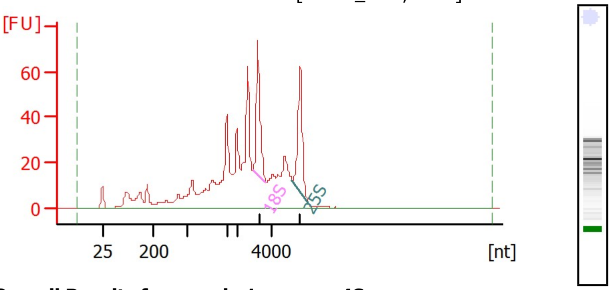

Overall Results for sample 1 : 4S

RNA Area:	801.8
RNA Concentration:	565 ng/µl
rRNA Ratio [25s / 18s]:	1.0
RNA Integrity Number (RIN):	5.9 (B.02.08)
Result Flagging Color:	
Result Flagging Label:	RIN: 5.90

Fragment table for sample 1 : 4S

Name	Start Size [nt]	End Size [nt]	Area	% of total Area
18S	2,879	3,688	72.1	9.0
25S	5,209	6,141	74.6	9.3

5S [Jansa_MBU, RNA]

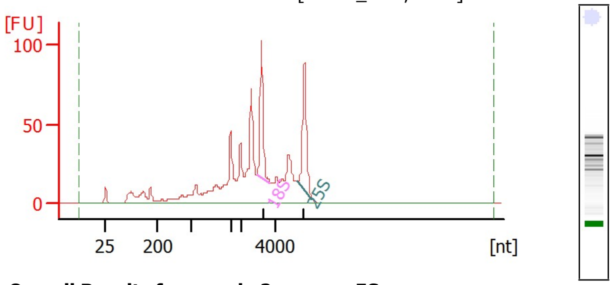

Overall Results for sample 2 : 5S

RNA Area:	875.4
RNA Concentration:	617 ng/µl
rRNA Ratio [25s / 18s]:	1.1
RNA Integrity Number (RIN):	6.1 (B.02.08)
Result Flagging Color:	
Result Flagging Label:	RIN: 6.10

Fragment table for sample 2 : 5S

Name	Start Size [nt]	End Size [nt]	Area	% of total Area
18S	2,869	3,673	85.5	9.8
25S	5,208	6,110	97.7	11.2

6S [Jansa_MBU, RNA]

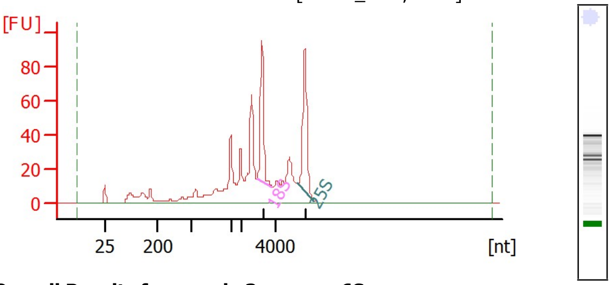

Overall Results for sample 3 : 6S

RNA Area:	747.6
RNA Concentration:	527 ng/µl
rRNA Ratio [25s / 18s]:	1.2
RNA Integrity Number (RIN):	6.4 (B.02.08)
Result Flagging Color:	
Result Flagging Label:	RIN: 6.40

Fragment table for sample 3 : 6S

Name	Start Size [nt]	End Size [nt]	Area	% of total Area
18S	2,860	3,683	86.6	11.6
25S	5,160	6,128	101.5	13.6

© Copyright 2003 - 2009 Agilent Technologies, Inc.

Assay Class: Plant RNA Nano
Data Path: D:\...00 expert_Plant RNA Nano_DE34904209_2015-04-22_12-16-22.xad

Created: 4/22/2015 12:16:22 PM
Modified: 4/22/2015 12:40:15 PM

Electropherogram Summary Continued ...

7S [Jansa_MBU, RNA]

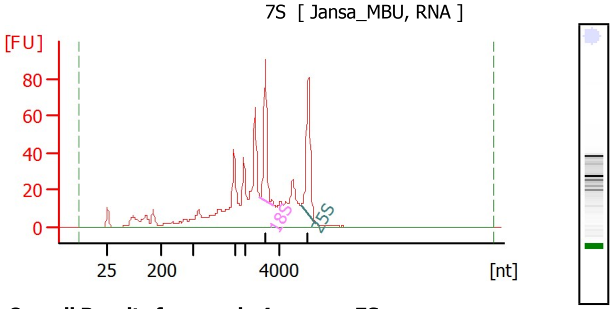

Overall Results for sample 4 : 7S

RNA Area: 776.2
RNA Concentration: 547 ng/µl
rRNA Ratio [25s / 18s]: 1.1
RNA Integrity Number (RIN): 6.1 (B.02.08)
Result Flagging Color:
Result Flagging Label: RIN: 6.10

Fragment table for sample 4 : 7S

Name	Start Size [nt]	End Size [nt]	Area	% of total Area
18S	2,870	3,665	77.8	10.0
25S	5,184	6,075	86.5	11.1

8S [Jansa_MBU, RNA]

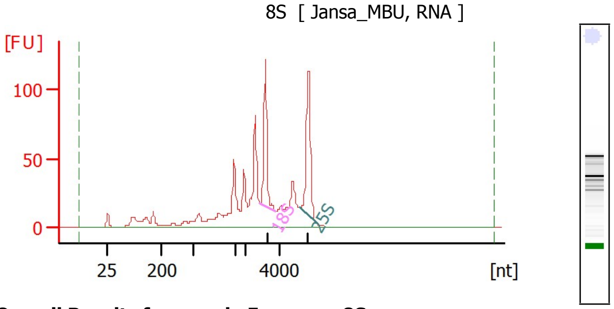

Overall Results for sample 5 : 8S

RNA Area: 894.9
RNA Concentration: 631 ng/µl
rRNA Ratio [25s / 18s]: 1.2
RNA Integrity Number (RIN): 6.4 (B.02.08)
Result Flagging Color:
Result Flagging Label: RIN: 6.40

Fragment table for sample 5 : 8S

Name	Start Size [nt]	End Size [nt]	Area	% of total Area
18S	2,846	3,689	106.7	11.9
25S	5,135	6,123	128.4	14.4

9S [Jansa_MBU, RNA]

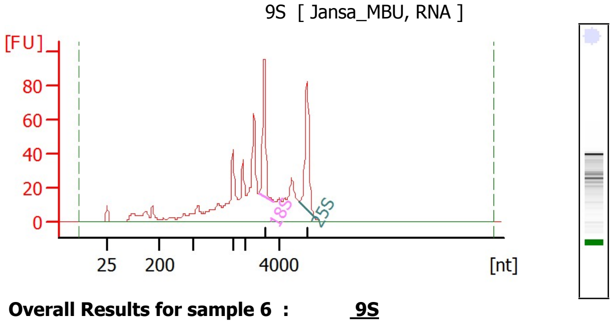

Overall Results for sample 6 : 9S

RNA Area: 784.8
RNA Concentration: 553 ng/µl
rRNA Ratio [25s / 18s]: 1.1
RNA Integrity Number (RIN): 6.1 (B.02.08)
Result Flagging Color:
Result Flagging Label: RIN: 6.10

Fragment table for sample 6 : 9S

Name	Start Size [nt]	End Size [nt]	Area	% of total Area
18S	2,841	3,710	80.0	10.2
25S	5,159	6,053	88.1	11.2

4R [Jansa_MBU, RNA]

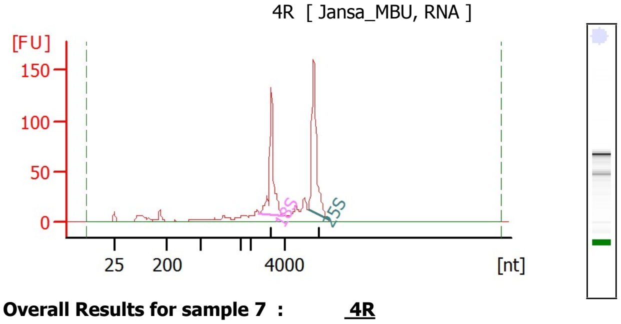

Overall Results for sample 7 : 4R

RNA Area: 698.1
RNA Concentration: 492 ng/µl
rRNA Ratio [25s / 18s]: 1.3
RNA Integrity Number (RIN): 9 (B.02.08)
Result Flagging Color:
Result Flagging Label: RIN:9

Fragment table for sample 7 : 4R

Name	Start Size [nt]	End Size [nt]	Area	% of total Area
18S	2,497	3,852	161.0	23.1
25S	5,281	6,442	210.7	30.2

 © Copyright 2003 - 2009 Agilent Technologies, Inc.

Assay Class: Plant RNA Nano
Data Path: D:\...00 expert_Plant RNA Nano_DE34904209_2015-04-22_12-16-22.xad

Created: 4/22/2015 12:16:22 PM
Modified: 4/22/2015 12:40:15 PM

Electropherogram Summary Continued ...

5R [Jansa_MBU, RNA]

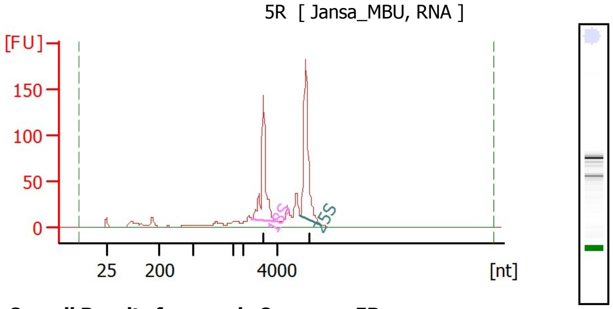

Overall Results for sample 8 : 5R

RNA Area:	744.3
RNA Concentration:	525 ng/µl
rRNA Ratio [25s / 18s]:	1.2
RNA Integrity Number (RIN):	9.2 (B.02.08)
Result Flagging Color:	
Result Flagging Label:	RIN: 9.20

Fragment table for sample 8 : 5R

Name	Start Size [nt]	End Size [nt]	Area	% of total Area
18S	2,497	3,877	186.3	25.0
25S	5,256	6,442	225.3	30.3

6R [Jansa_MBU, RNA]

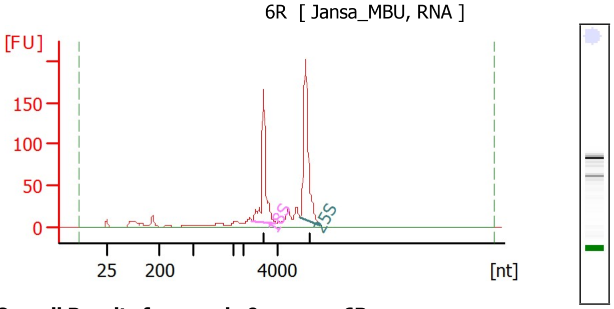

Overall Results for sample 9 : 6R

RNA Area:	789.3
RNA Concentration:	557 ng/µl
rRNA Ratio [25s / 18s]:	1.4
RNA Integrity Number (RIN):	9.4 (B.02.08)
Result Flagging Color:	
Result Flagging Label:	RIN: 9.40

Fragment table for sample 9 : 6R

Name	Start Size [nt]	End Size [nt]	Area	% of total Area
18S	2,497	3,852	193.0	24.5
25S	5,232	6,394	269.3	34.1

7R [Jansa_MBU, RNA]

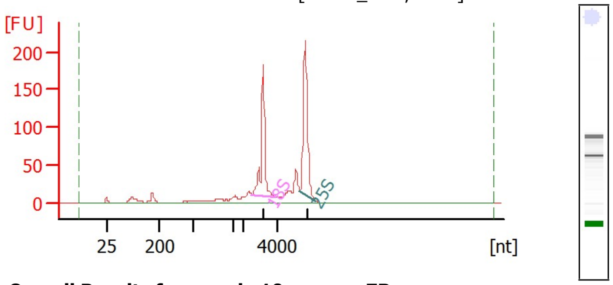

Overall Results for sample 10 : 7R

RNA Area:	864.7
RNA Concentration:	610 ng/µl
rRNA Ratio [25s / 18s]:	1.2
RNA Integrity Number (RIN):	8.9 (B.02.08)
Result Flagging Color:	
Result Flagging Label:	RIN: 8.90

Fragment table for sample 10 : 7R

Name	Start Size [nt]	End Size [nt]	Area	% of total Area
18S	2,473	3,852	203.0	23.5
25S	5,232	6,176	251.7	29.1

8R [Jansa_MBU, RNA]

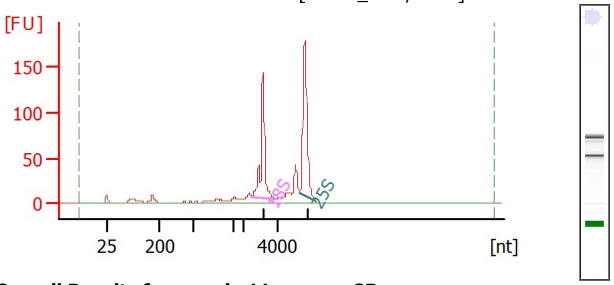

Overall Results for sample 11 : 8R

RNA Area:	662.8
RNA Concentration:	467 ng/µl
rRNA Ratio [25s / 18s]:	1.3
RNA Integrity Number (RIN):	9.3 (B.02.08)
Result Flagging Color:	
Result Flagging Label:	RIN: 9.30

Fragment table for sample 11 : 8R

Name	Start Size [nt]	End Size [nt]	Area	% of total Area
18S	2,449	3,852	166.7	25.2
25S	5,208	6,176	213.1	32.2

 © Copyright 2003 - 2009 Agilent Technologies, Inc.

Assay Class: Plant RNA Nano
Data Path: D:\...00 expert_Plant RNA Nano_DE34904209_2015-04-22_12-16-22.xad

Created: 4/22/2015 12:16:22 PM
Modified: 4/22/2015 12:40:15 PM

Electropherogram Summary Continued ...

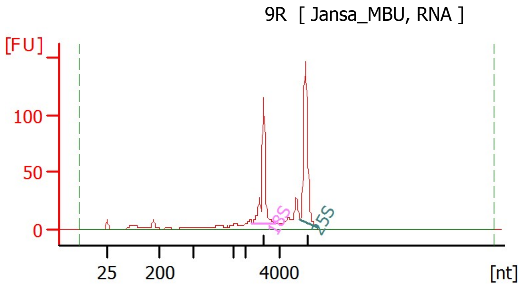

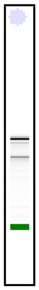

Overall Results for sample 12 : 9R

RNA Area:	526.1
RNA Concentration:	371 ng/µl
rRNA Ratio [25s / 18s]:	1.4
RNA Integrity Number (RIN):	9.2 (B.02.08)
Result Flagging Color:	
Result Flagging Label:	RIN: 9.20

Fragment table for sample 12 : 9R

Name	Start Size [nt]	End Size [nt]	Area	% of total Area
18S	2,455	3,807	123.6	23.5
25S	5,184	6,174	169.3	32.2

 © Copyright 2003 - 2009 Agilent Technologies, Inc.

Assay Class: Plant RNA Nano
Data Path: D:\...00 expert_Plant RNA Nano_DE34904209_2015-04-22_12-16-22.xad
Created: 4/22/2015 12:16:22 PM
Modified: 4/22/2015 12:40:15 PM

Gel Image

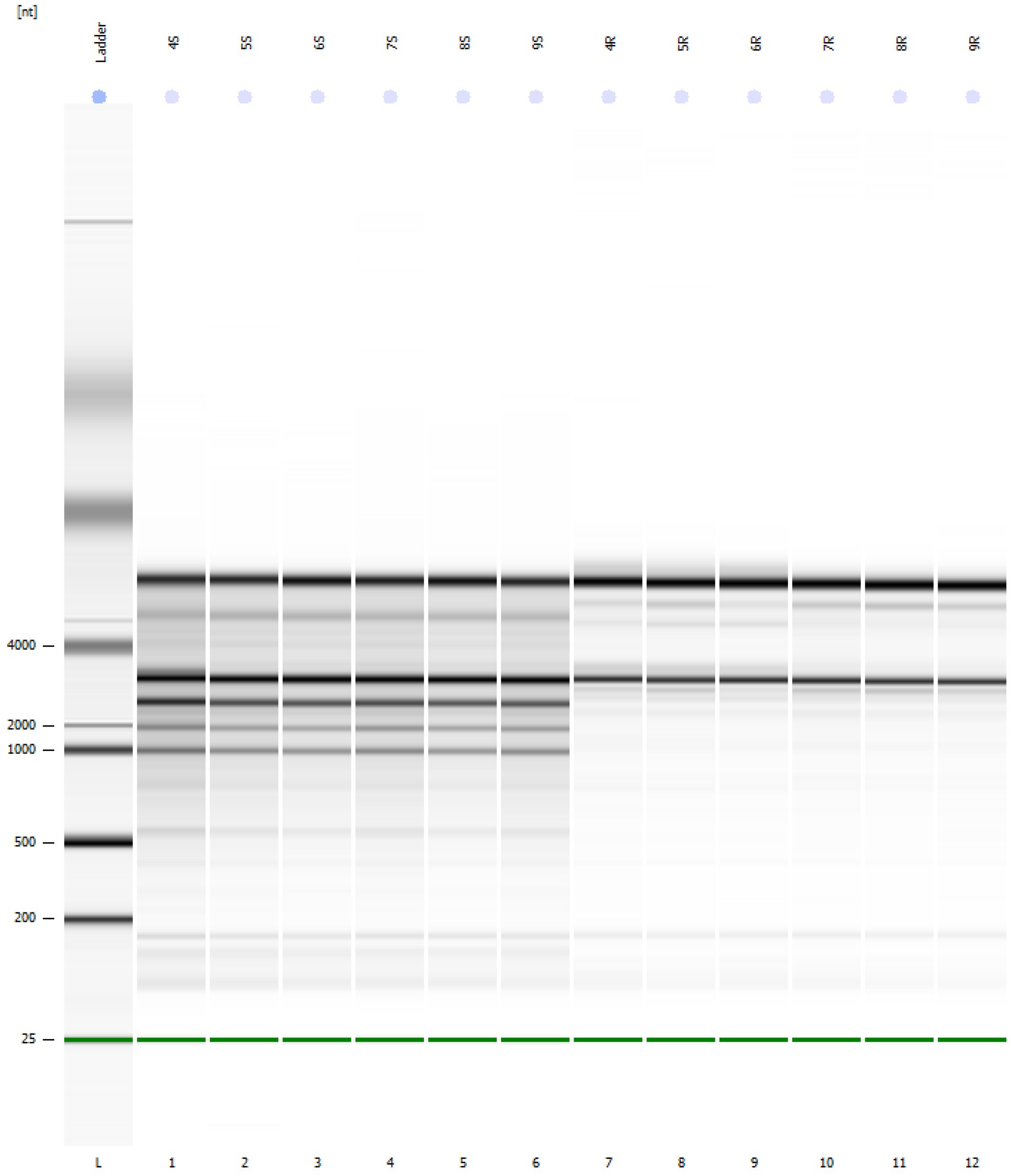

 © Copyright 2003 - 2009 Agilent Technologies, Inc.

Assay Class: Plant RNA Nano
Data Path: D:\...00 expert_Plant RNA Nano_DE34904209_2015-04-22_12-16-22.xad

Created: 4/22/2015 12:16:22 PM
Modified: 4/22/2015 12:40:15 PM

Run Logbook

Description	Number	Source	Category	Sub Category	Time	Time Zone	User	Host
Run ended on port 1 (Number of wells acquired: 13)		Instrument	Run		4/22/2015 12:40:13 PM	(GMT +02:00) Central Europe Standard Time	corelab	NAT4AGILENT
Run started on port 1 (File: D:\Agilent\data\2015-04-22\2100 expert_Plant RNA Nano_DE34904209_2015-04-22_12-16-22.xad)		Instrument	Run		4/22/2015 12:16:27 PM	(GMT +02:00) Central Europe Standard Time	corelab	NAT4AGILENT
Product Number : G2938C		Instrument	Run		4/22/2015 12:16:27 PM	(GMT +02:00) Central Europe Standard Time	corelab	NAT4AGILENT
Name : IMG-CORE-AGILENT		Instrument	Run		4/22/2015 12:16:27 PM	(GMT +02:00) Central Europe Standard Time	corelab	NAT4AGILENT
Vendor : Agilent Technologies		Instrument	Run		4/22/2015 12:16:27 PM	(GMT +02:00) Central Europe Standard Time	corelab	NAT4AGILENT
Serial# : DE34904209		Instrument	Run		4/22/2015 12:16:27 PM	(GMT +02:00) Central Europe Standard Time	corelab	NAT4AGILENT
Firmware : C.01.069		Instrument	Run		4/22/2015 12:16:27 PM	(GMT +02:00) Central Europe Standard Time	corelab	NAT4AGILENT
Cartridge : Electrode		Instrument	Run		4/22/2015 12:16:27 PM	(GMT +02:00) Central Europe Standard Time	corelab	NAT4AGILENT

© Copyright 2003 - 2009 Agilent Technologies, Inc.
