# Supplementary figures and images for: Correlative evidence for co-regulation of phosphorus and carbon exchanges with symbiotic fungus in the arbuscular mycorrhizal Medicago truncatula
Source: PLoS One. 2019 Nov 11;14(11):e0224938. doi: 10.1371/journal.pone.0224938 (PMC6844471; doi:10.1371/journal.pone.0224938)

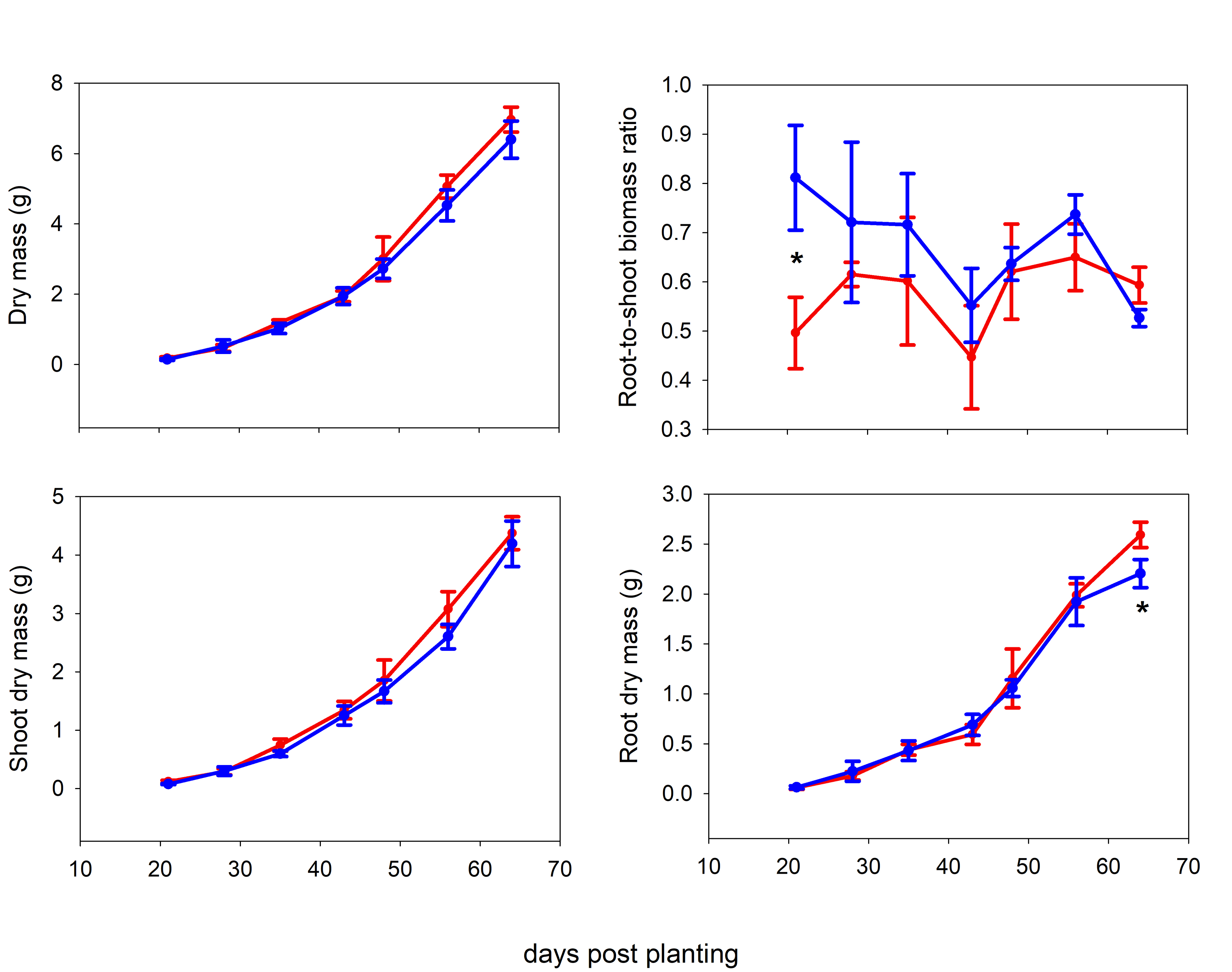

Supplement: S3 Fig — (A) total dry mass of whole plants per pot; (B) root to shoot ratio; (C) dry mass of whole shoots per pot; (D) dry mass of whole roots per pot. Error bars show standard deviations, n = 3. Red: mycorrhizal, Blue: non-mycorrhizal plants. Asterisks indicate significance levels as per t-test comparing mycorrhizal and non-mycorrhizal treatments at the different timepoints: 0.01 ≤ * < 0.05. When no asterisk is displayed, the values did not significantly differ between the mycorrhizal and non-mycorrhizal treatments (i.e., p ≥ 0.05). (TIF) [file pone.0224938.s006.tif]

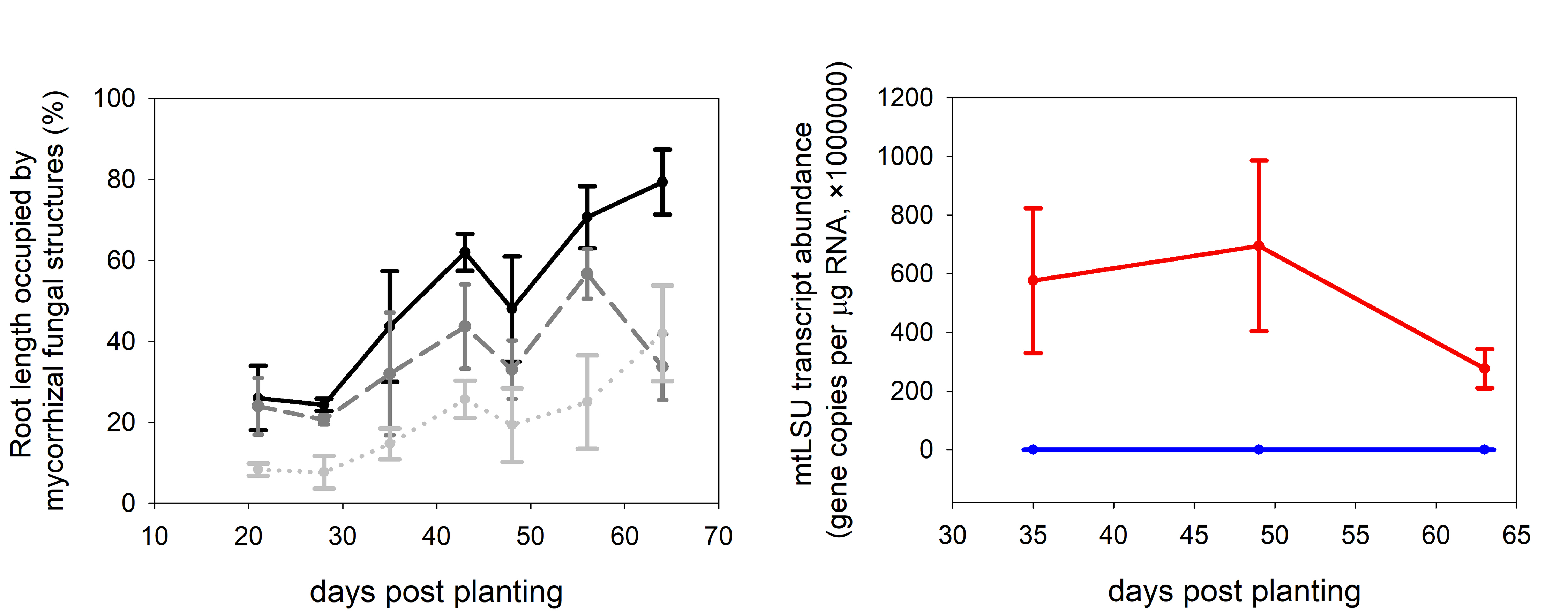

Supplement: S4 Fig — Colonization of the plant roots measured by magnified intersection method following staining of the roots (left panel) as described previously [61], with the solid line and black color indicating root occupancy by the fungal hyphae, dashed line and dark gray color standing for arbuscules, and dotted line and light gray color standing for vesicles (non-mycorrhizal root samples did not show any colonization and the results are thus not displayed), and by quantitative real-time PCR (right panel) using the mt5 marker set [62]. Red: mycorrhizal, Blue: non-mycorrhizal plants. Mean values of 3 replicate values per each timepoint are shown, error bars indicate ± standard deviations. (TIF) [file pone.0224938.s007.tif]

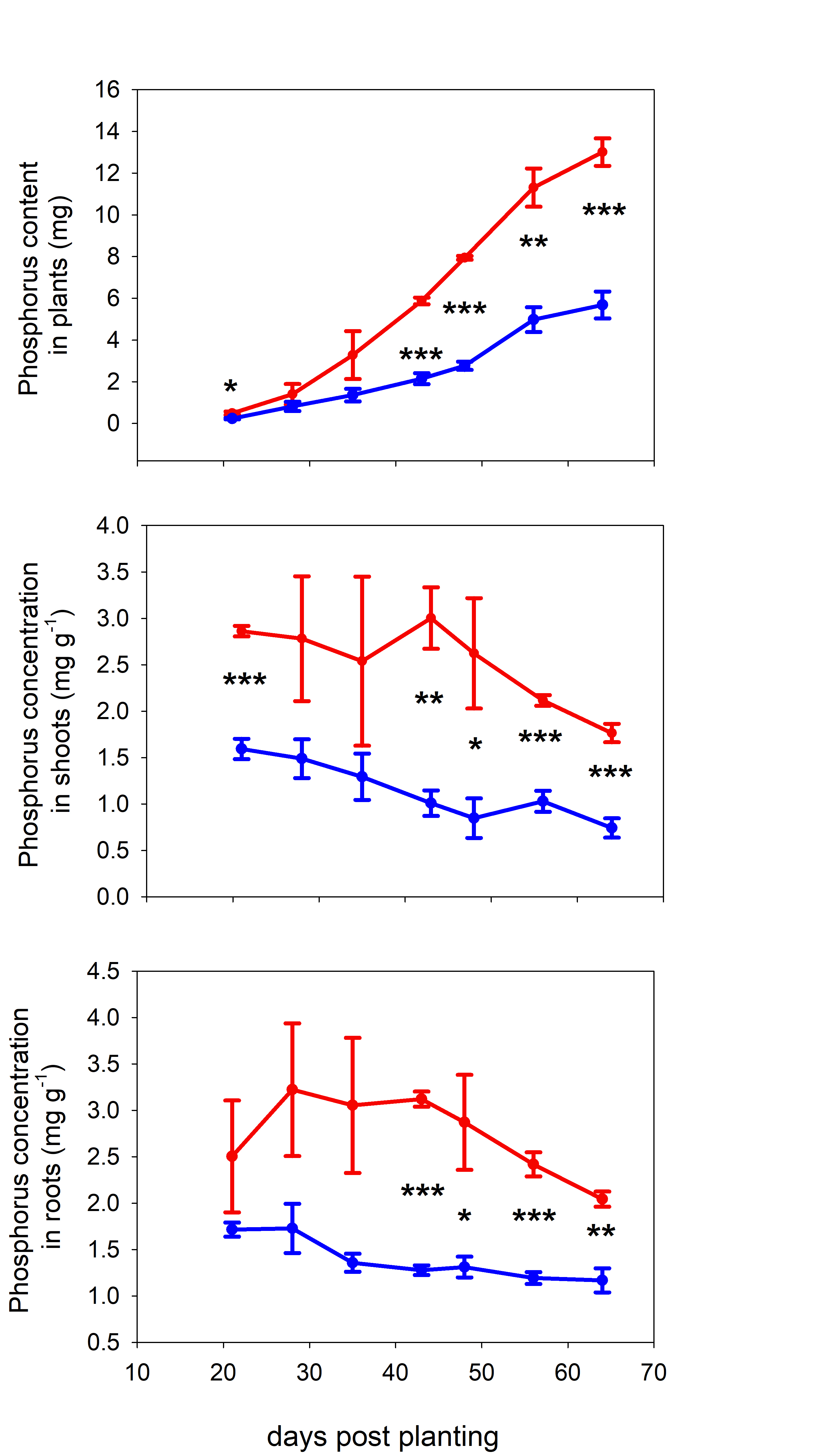

Supplement: S5 Fig — Total phosphorus content in whole plants per pot (upper panel), phosphorus concentration in the shoots (middle panel) and phosphorus concentration in the roots (lower panel). Error bars show standard deviation, n = 3. Red: mycorrhizal, Blue: non-mycorrhizal plants. Asterisks indicate significance levels as per t-test comparing mycorrhizal and non-mycorrhizal treatments at the different timepoints: 0 < *** < 0.001 ≤ ** < 0.01 ≤ * < 0.05. When no asterisk is displayed, the values did not significantly differ between the mycorrhizal and non-mycorrhizal treatments (i.e., p ≥ 0.05). (TIF) [file pone.0224938.s008.tif]

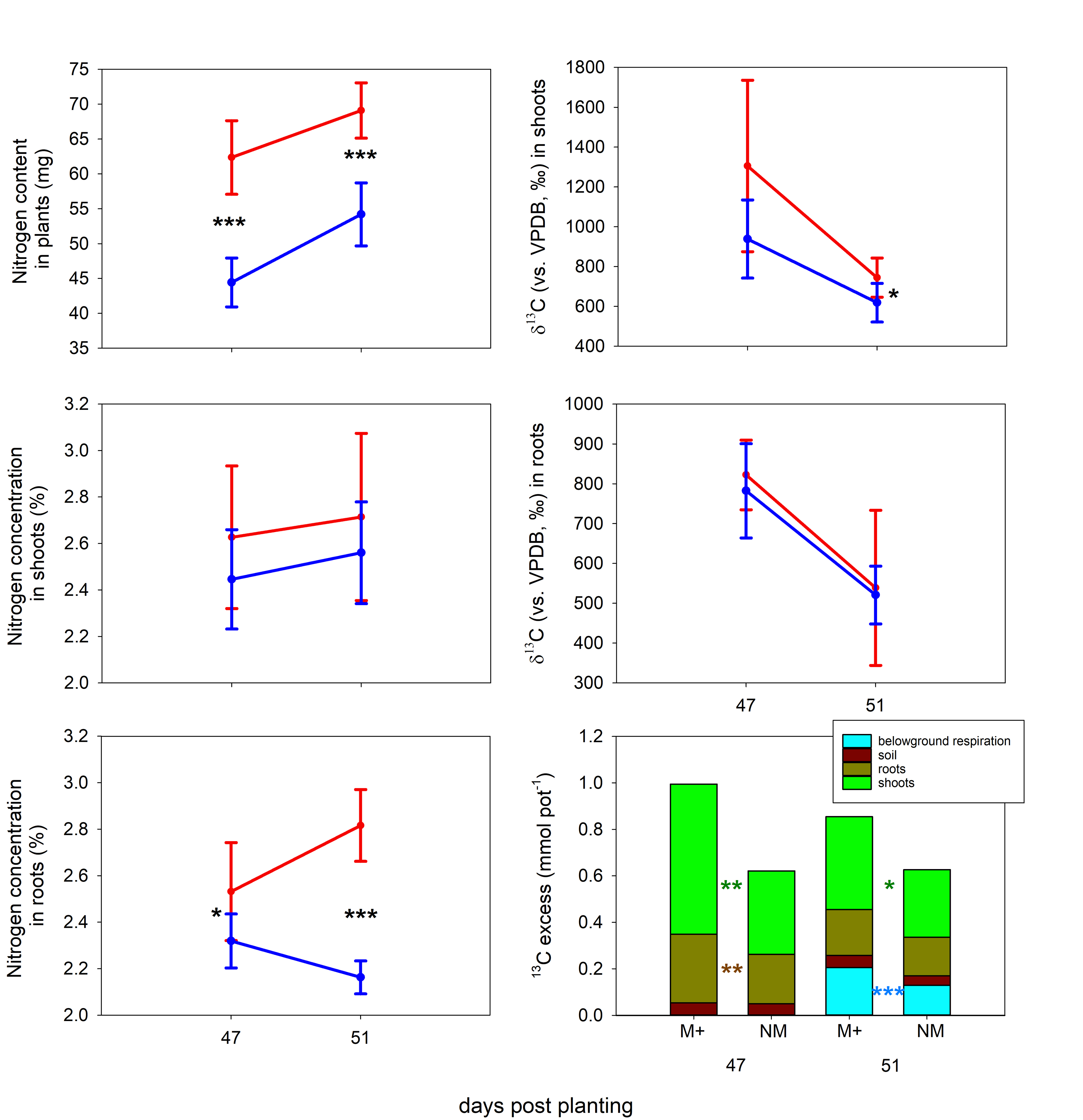

Supplement: S6 Fig — Carbon isotopic composition (right panels) and nitrogen concentrations (left panels) measured just after (within 15 min) and 4 days after pulse-labeling the plants with 13CO2 at 47 dpp, using the experimental framework described in Slavíková et al. [63]. Error bars show standard deviations, n = 7. Asterisks indicate significance levels as per t-test comparing mycorrhizal (red or M+) and non-mycorrhizal (blue or NM) treatments at the different timepoints, or between the groups: 0 < *** < 0.001 ≤ ** < 0.01 ≤ * < 0.05. When no asterisk is displayed, the values did not significantly differ between the mycorrhizal and non-mycorrhizal treatments or the other sample groups (i.e., p ≥ 0.05). (TIF) [file pone.0224938.s009.tif]

Photo 1

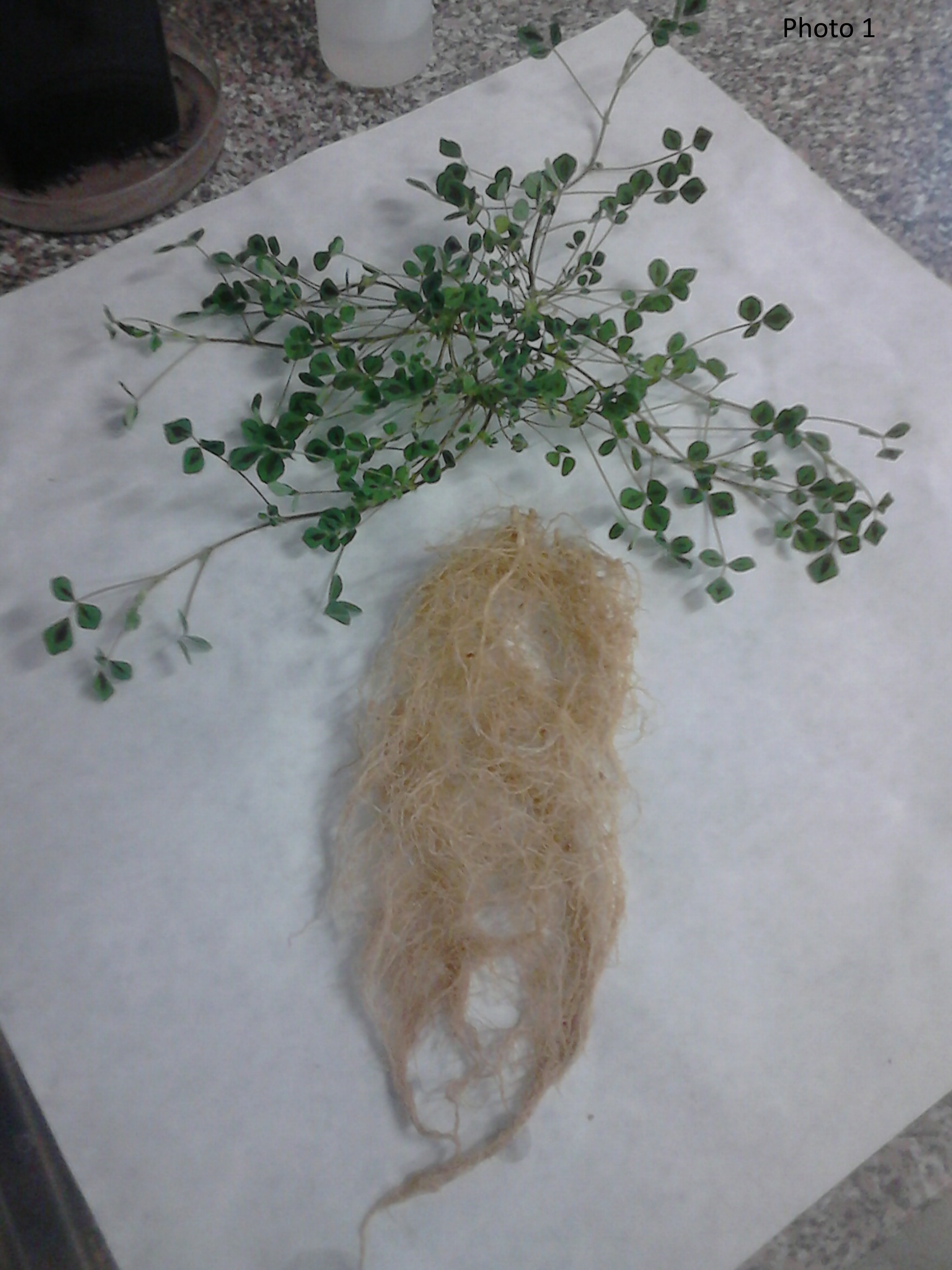

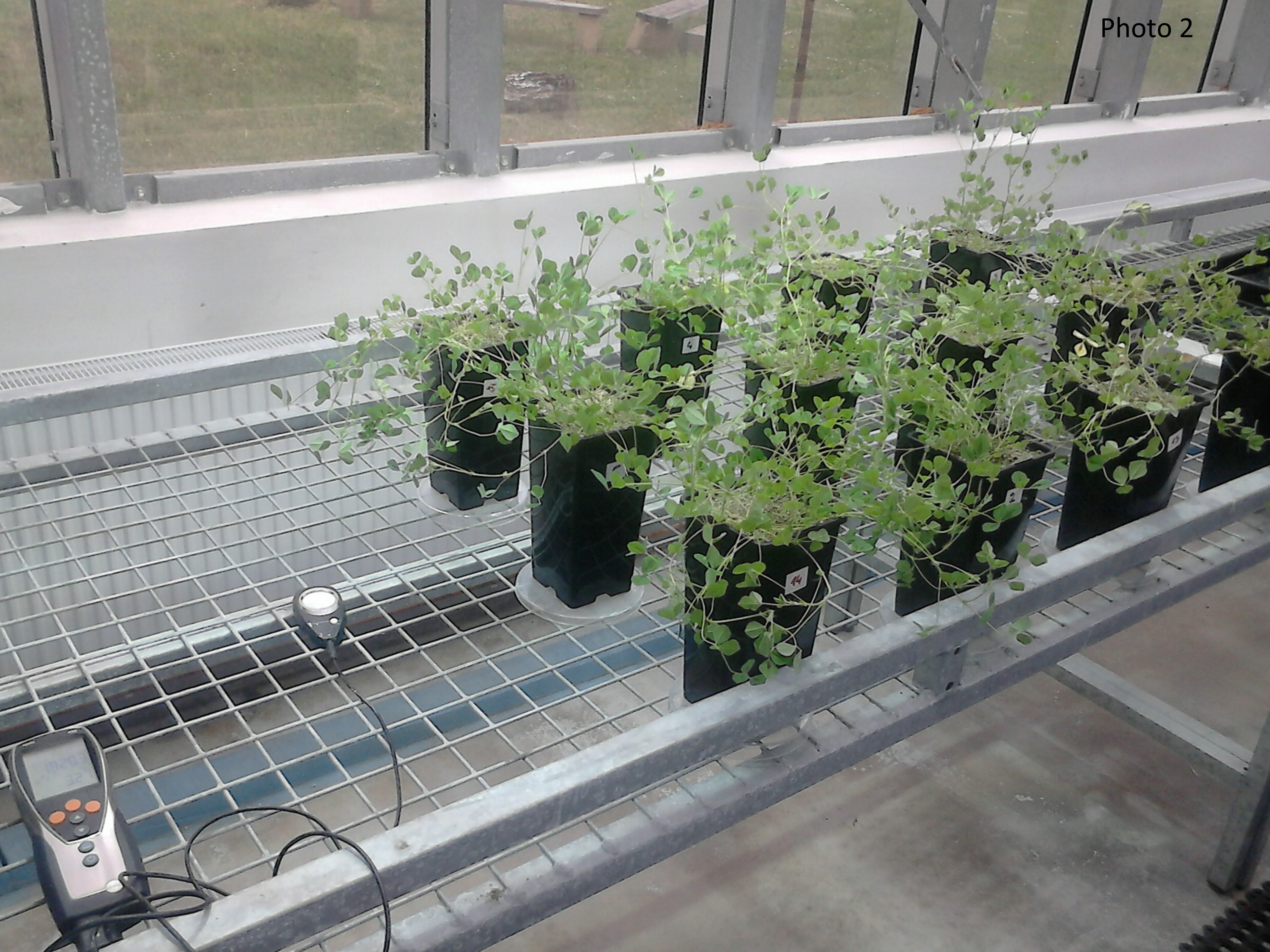

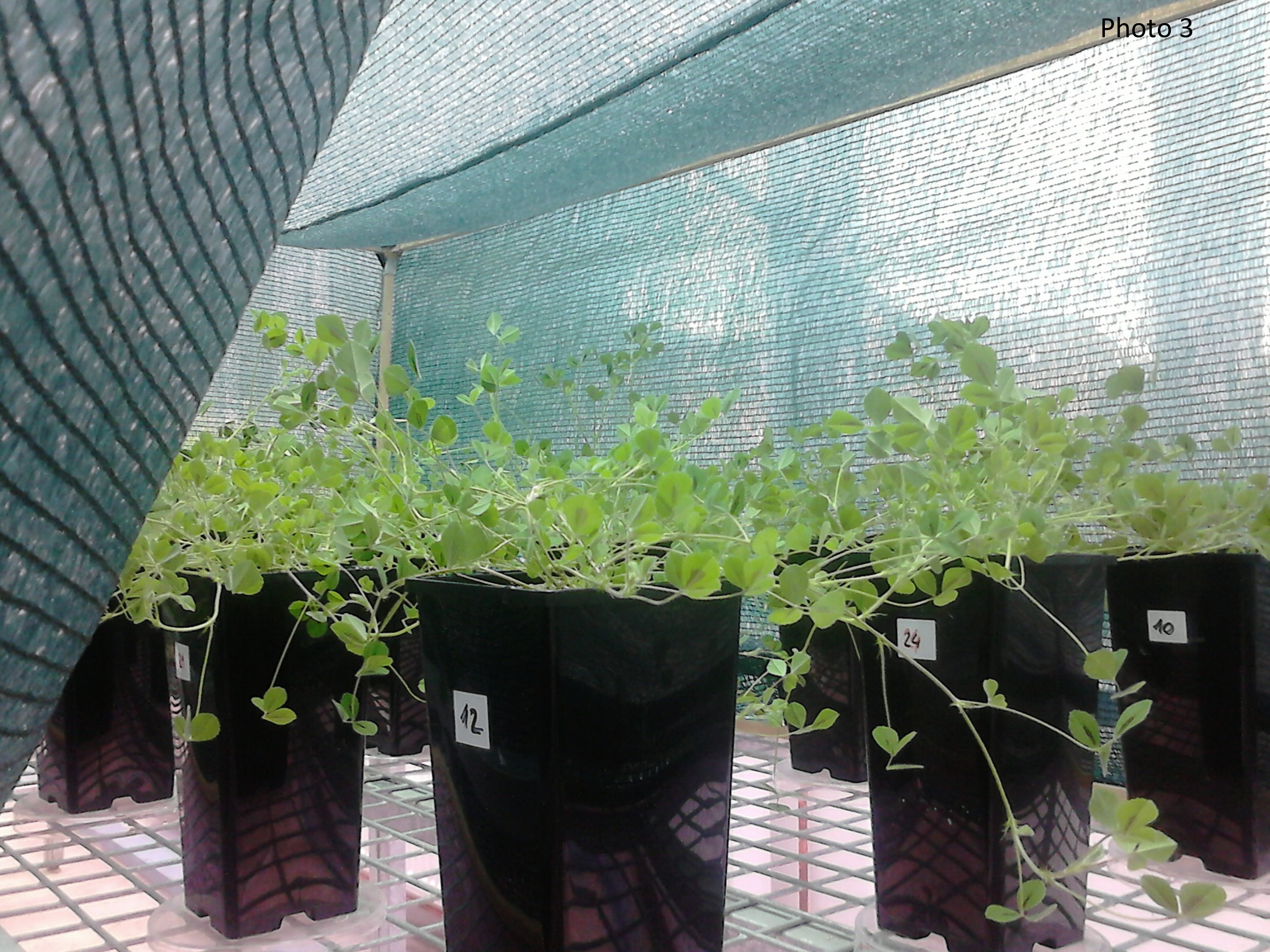

Supplement: S1 Photos — Photo 1: M. truncatula plants harvested from one pot at 45 dpp (Exp 1)–examples of whole root and whole shoot samples. Photo 2: Non-shaded M. truncatula plants in pot cultures during growth at 64 dpp (Exp 2). Photo 3: Shaded M. truncatula plants in pot cultures during growth at 64 dpp (Exp 2). (PDF) [file pone.0224938.s011.pdf]
