# Supplementary material for: Correlative evidence for co-regulation of phosphorus and carbon exchanges with symbiotic fungus in the arbuscular mycorrhizal Medicago truncatula
Source: PLoS One. 2019 Nov 11;14(11):e0224938. doi: 10.1371/journal.pone.0224938 (PMC6844471; doi:10.1371/journal.pone.0224938)
Supplement: S7 Fig — The expression of genes measured by the quantitative real-time PCR in M. truncatula roots (A and C) or shoots (B and D) in Exp 1 (A and B) or Exp 2 (C and D), respectively. X-axis: days post planting, Y-axis: number of corresponding-gene transcript copies measured per 1 μg of RNA subjected to reverse transcription. Red: mycorrhizal treatment, Blue: non-mycorrhizal treatment; Full line: full light (100%), dashed-line: shaded plants (10% of light). Error bars show standard deviations, n = 6 or 3 for Exp 1 or Exp 2, respectively. For further details see Materials and methods and S2 & S3 Tables. Asterisks indicate significance levels as per t-test comparing mycorrhizal (red) and non-mycorrhizal (blue) treatments (Exp 1) or non-shaded and shaded plants within the mycorrhizal or non-mycorrhizal treatments (Exp 2) at the different timepoints: 0 < *** < 0.001 ≤ ** < 0.01 ≤ * < 0.05. When no asterisk is displayed, the values did not significantly differ between the mycorrhizal and non-mycorrhizal treatments (i.e., p ≥ 0.05). (PDF) [file pone.0224938.s010.pdf]

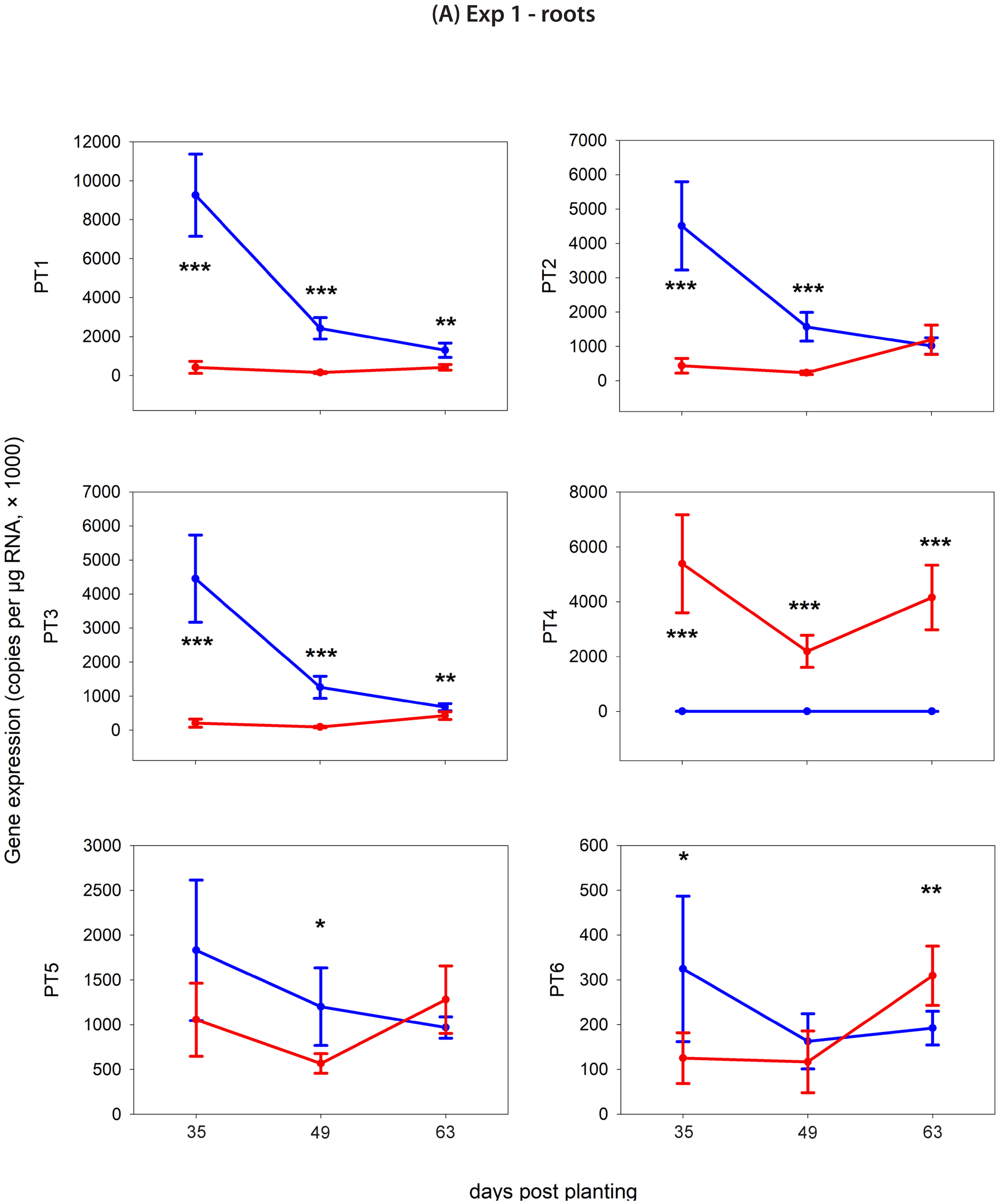
(A) Exp 1 - roots
PT1
PT2
PT3
PT4
PT5
PT6
***
**
*
Gene expression (copies per µg RNA, × 1000)
35
49
63
days post planting

(A) Exp 1 - roots

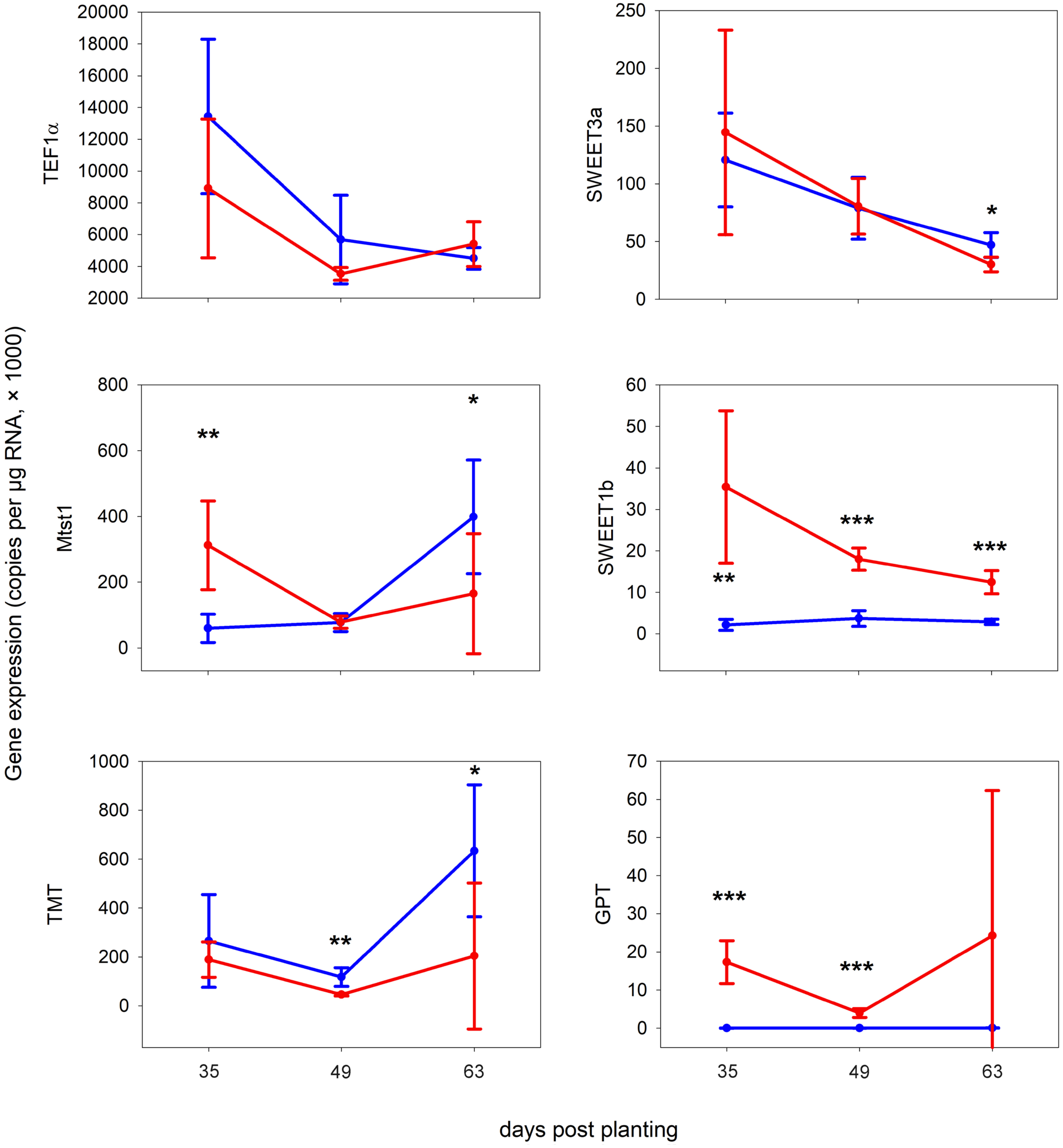

Gene expression (copies per µg RNA, × 1000)
TEF1α
20000
18000
16000
14000
12000
10000
8000
6000
4000
2000
SWEET3a
250
200
150
100
50
0
*
Mtst1
800
600
400
200
0
**
*
SWEET1b
60
50
40
30
20
10
0
**
***
***
TMT
1000
800
600
400
200
0
**
*
GPT
70
60
50
40
30
20
10
0
***
***
35
49
63
35
49
63
days post planting

(A) Exp 1 - roots

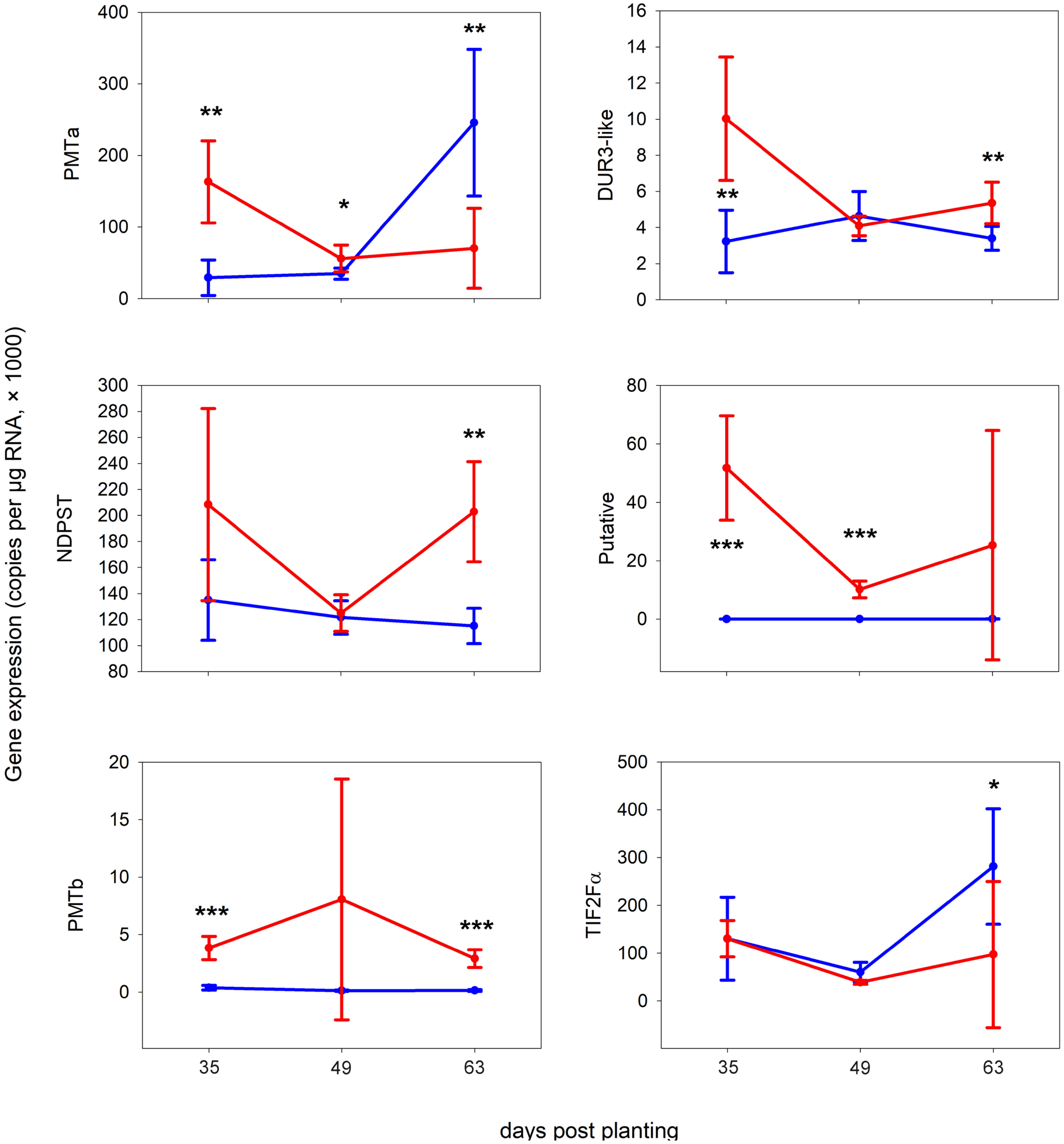

PMTa
DUR3-like
NDPST
Putative
PMTb
TIF2Fα
Gene expression (copies per µg RNA, × 1000)
days post planting
35
49
63

(B) Exp 1 - shoots

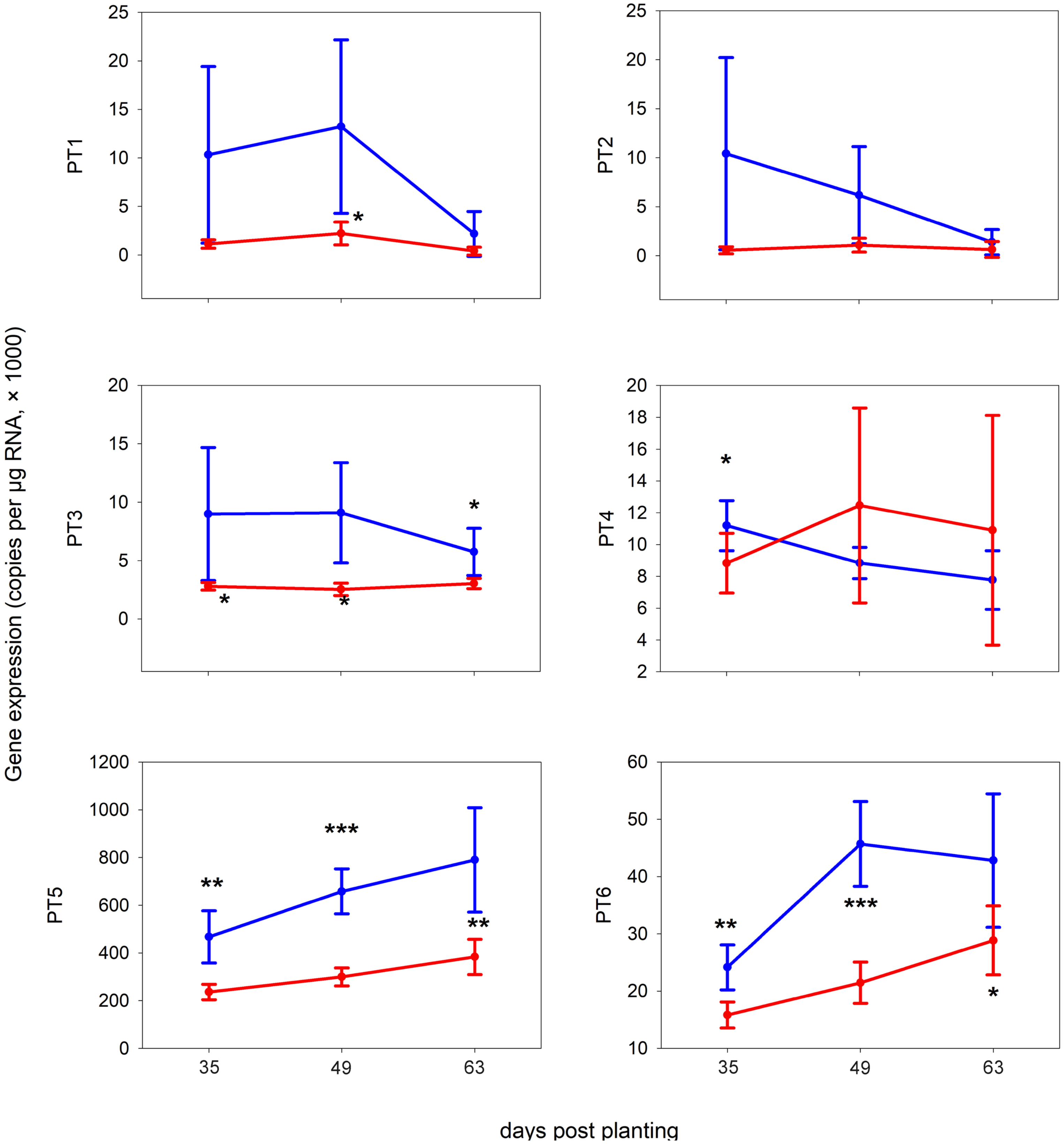

(B) Exp 1 - shoots

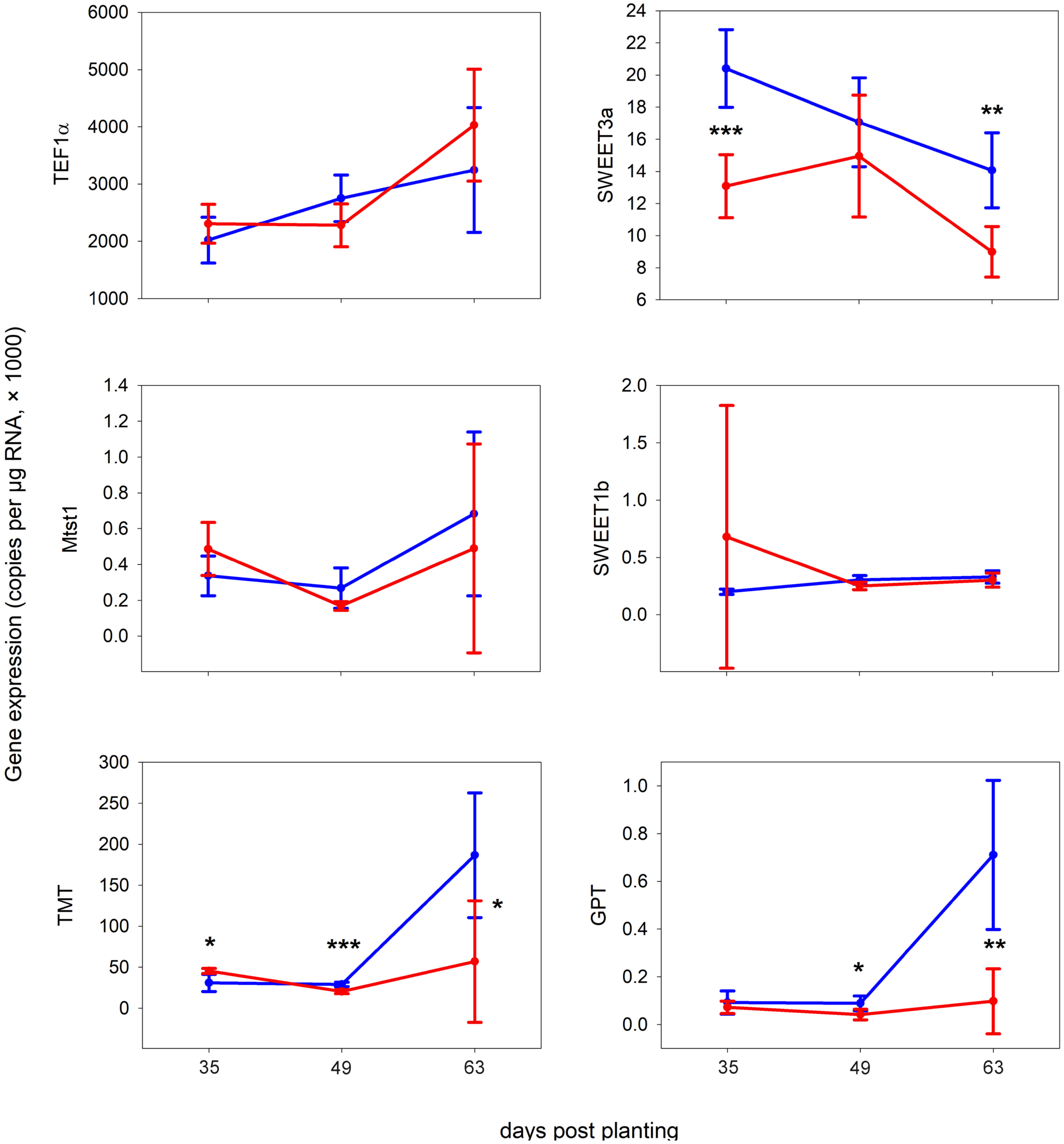

(B) Exp 1 - shoots

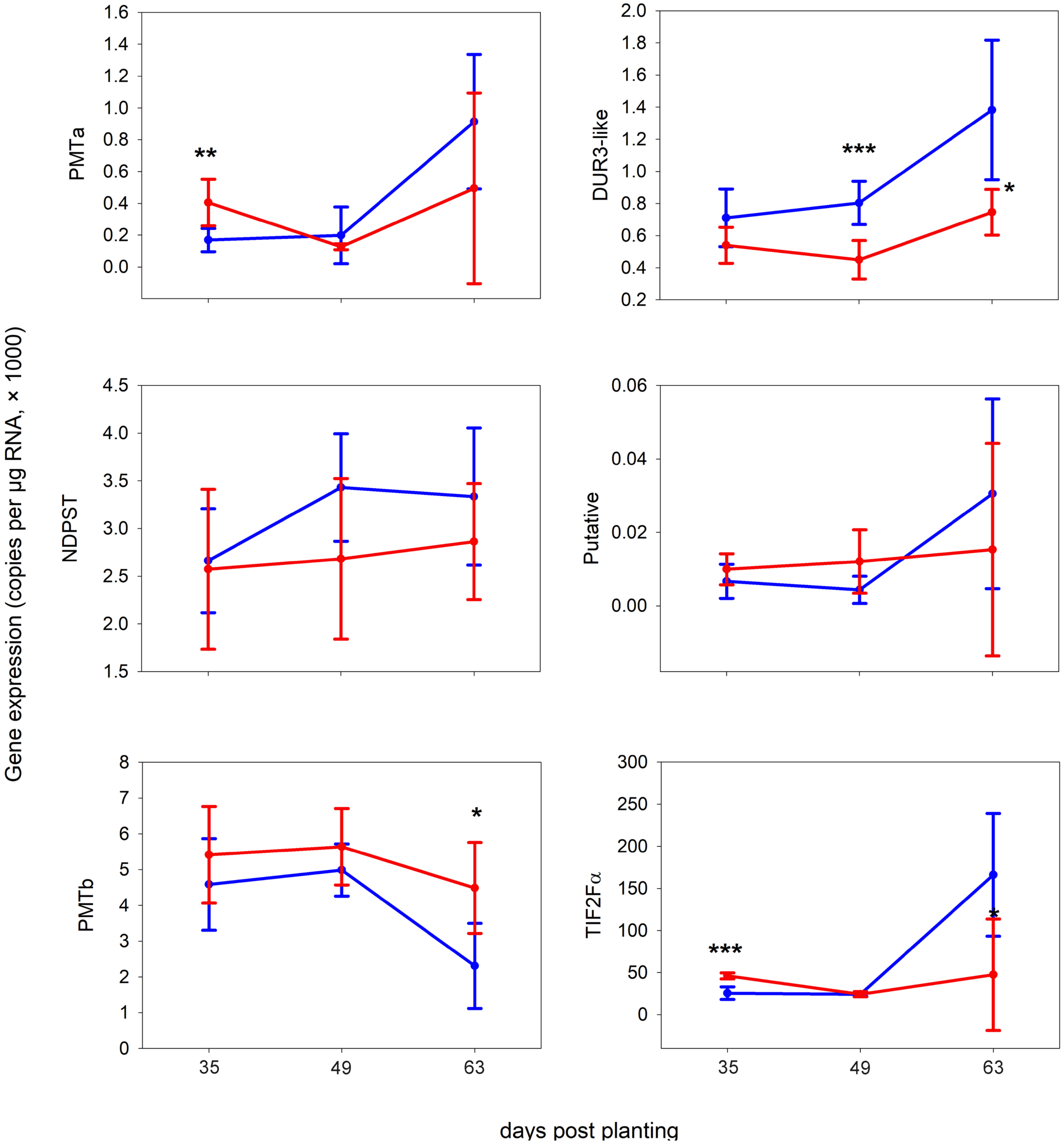

(C) Exp 2 - roots

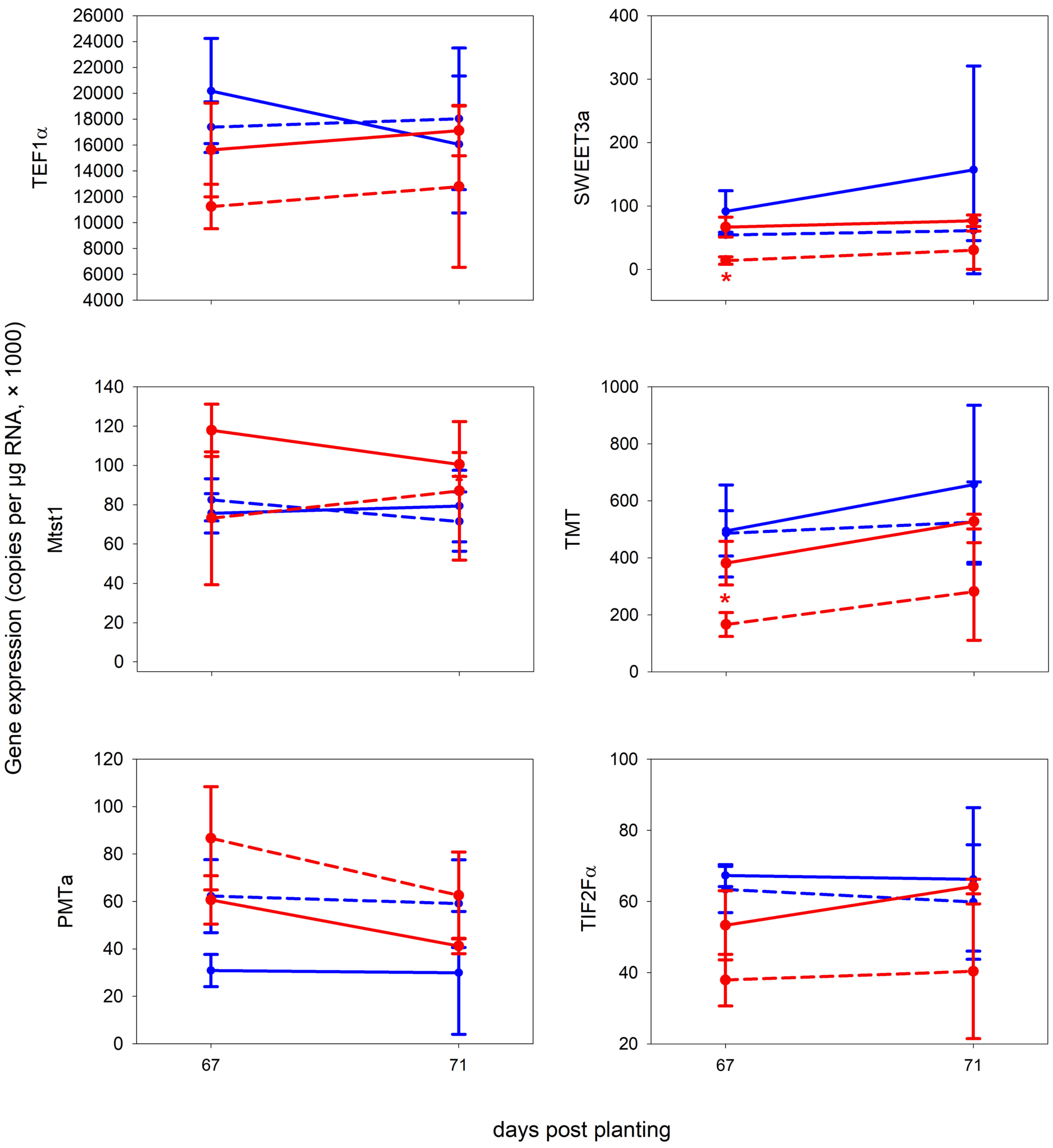

Gene expression (copies per µg RNA, × 1000)
TEF1α
SWEET3a
Mtst1
TMT
PMTa
TIF2Fα
67
71
days post planting

(D) Exp 2 - shoots

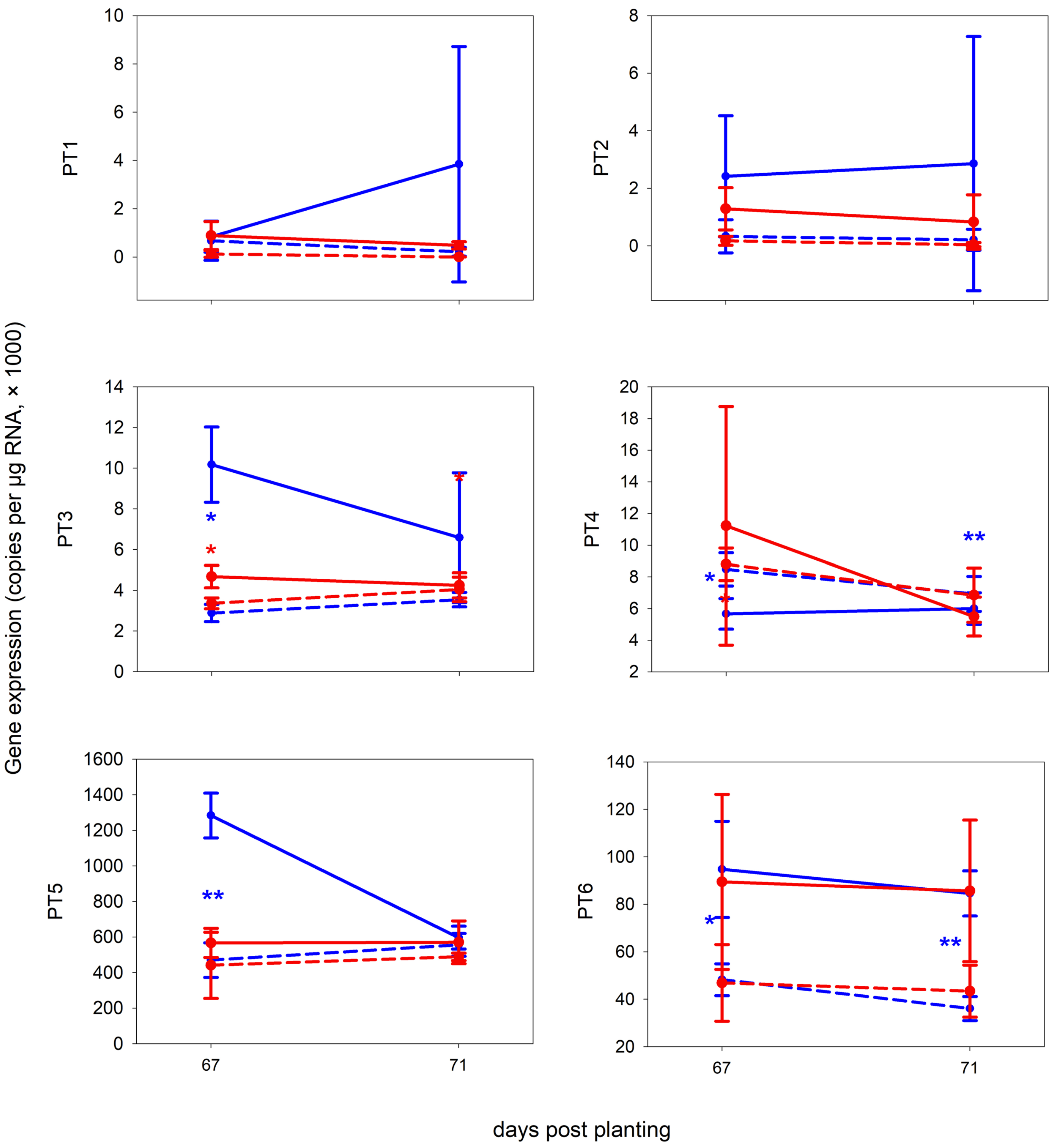

(D) Exp 2 - shoots

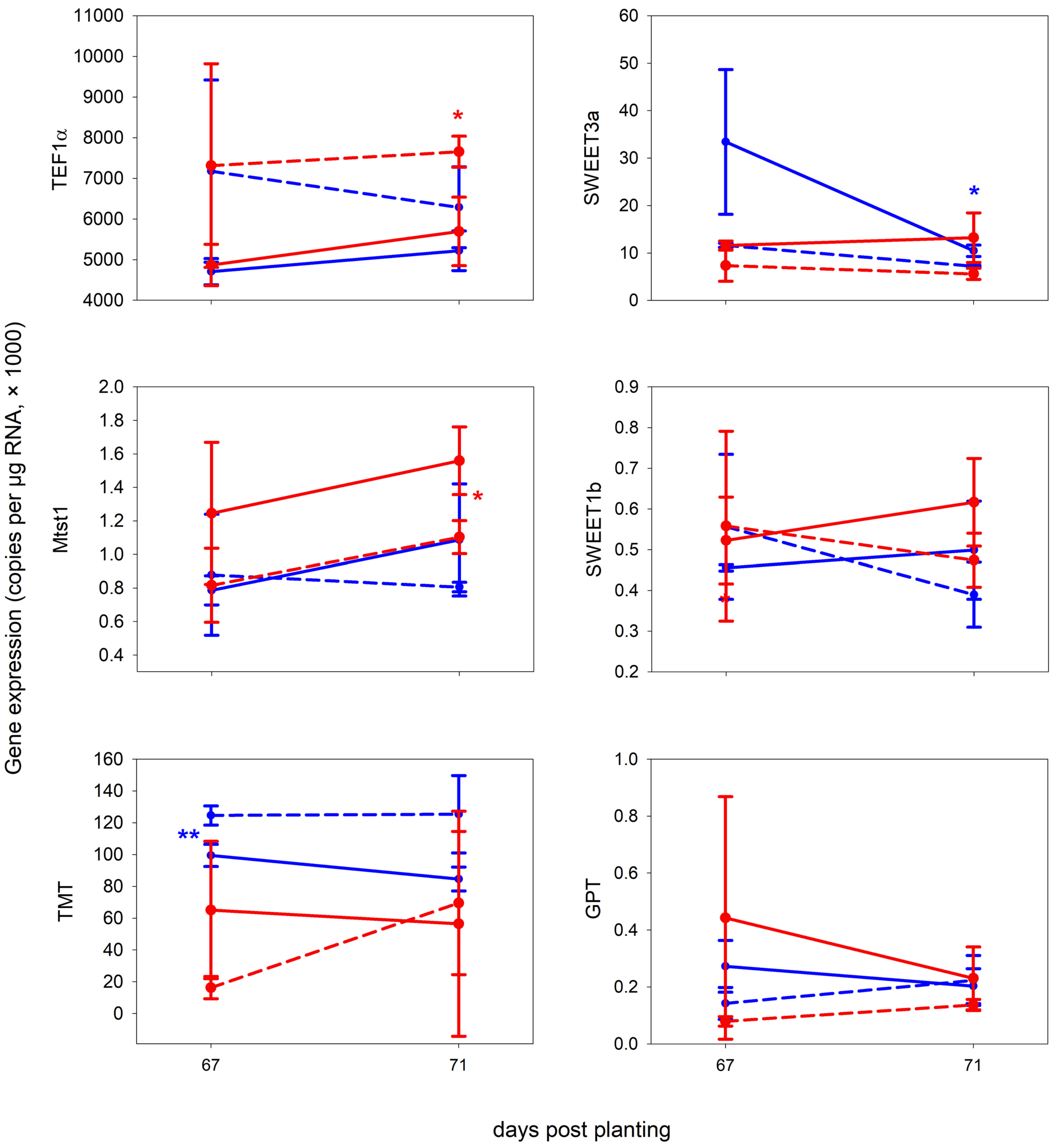

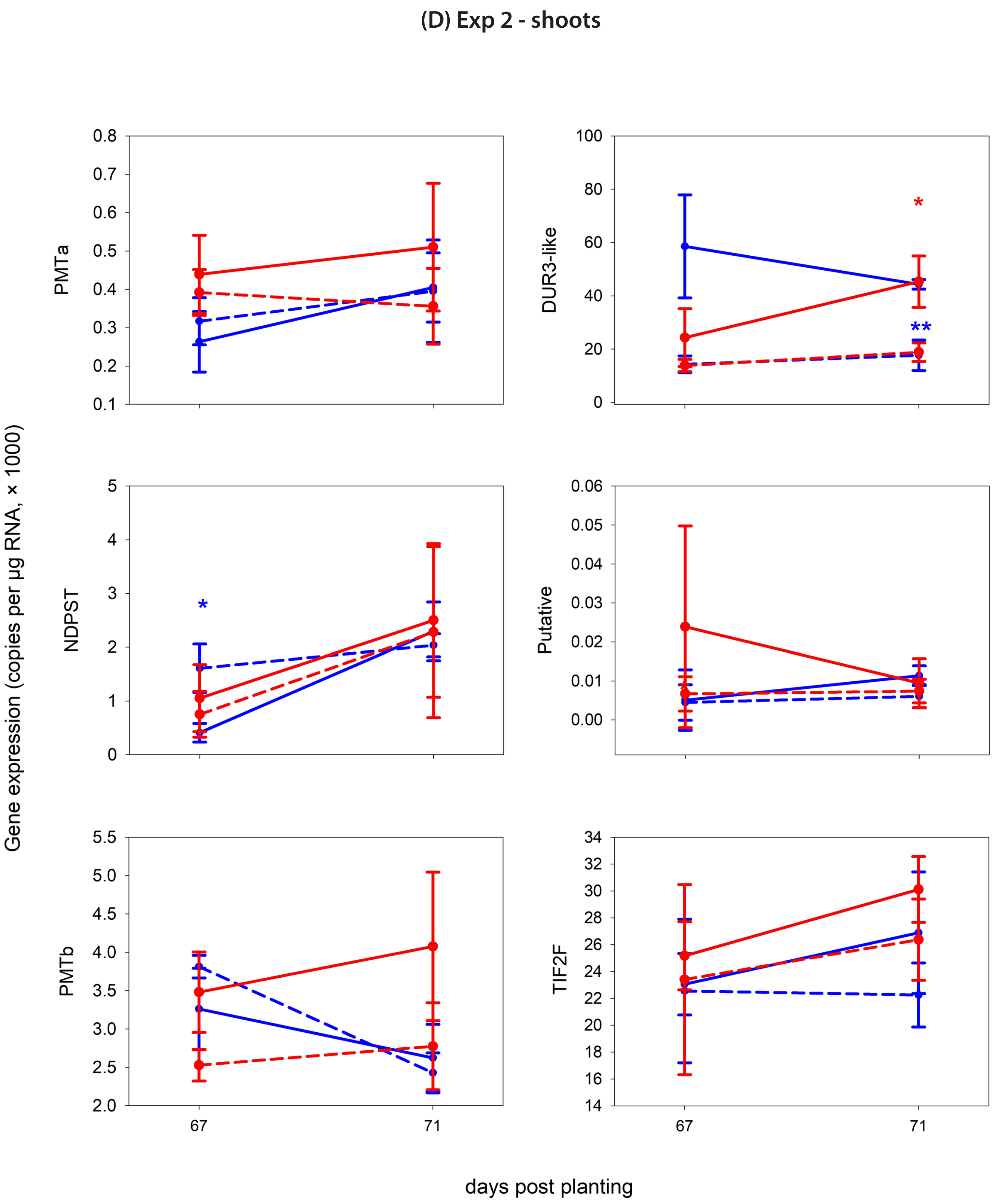

(D) Exp 2 - shoots
Gene expression (copies per µg RNA, × 1000)
PMTa
DUR3-like
NDPST
Putative
PMTb
TIF2F
*
**
*
67
71
days post planting
